# Changes in healthcare seeking and lifestyle in old aged individuals during COVID-19 lockdown in Germany: the population-based AugUR study

**DOI:** 10.1186/s12877-021-02677-x

**Published:** 2022-01-08

**Authors:** Caroline Brandl, Martina E. Zimmermann, Felix Günther, Alexander Dietl, Helmut Küchenhoff, Julika Loss, Klaus J. Stark, Iris M. Heid

**Affiliations:** 1 Department of Genetic Epidemiology, University of Regensburg, Franz-Josef-Strauß-Alle 11, 93053 Regensburg, Germany; 2 Department of Ophthalmology, University Hospital Regensburg, Regensburg, Germany; 3 Institute of Human Genetics, University of Regensburg, Regensburg, Germany; 4 Statistical Consulting Unit StaBLab, Department of Statistics, Ludwig-Maximilians-University Munich, Munich, Germany; 5 Department of Internal Medicine II, University Hospital Regensburg, Regensburg, Germany; 6 Medical Sociology, Department for Epidemiology and Preventive Medicine, University of Regensburg, Dr. Gessler-Str. 17, 93051 Regensburg, Germany; 7 Robert Koch-Institut, Abteilung für Epidemiologie und Gesundheitsmonitoring, Berlin, Germany

**Keywords:** SARS-CoV-2, Coronavirus, COVID-19, AugUR, Population-based study, Old aged population, Lifestyle factors, Quality of life, Proportion at risk for COVID-19, Physical activity, smoking, alcohol consumption

## Abstract

**Background:**

Containment measures in the COVID-19 pandemic protected individuals at high risk, particularly individuals at old age, but little is known about how these measures affected health-related behavior of old aged individuals. We aimed to investigate the impact of the spring 2020 lockdown in Germany on healthcare-seeking and health-related lifestyle in the old aged and to identify susceptible subgroups.

**Methods:**

We conducted a follow-up survey among the pre-pandemically well-characterized participants of our AugUR cohort study, residents in/around Regensburg aged 70+ years and relatively mobile. A self-completion questionnaire on current behavior, perceived changes, and SARS-Cov-2 infection was mailed in May 2020, shortly before contact restrictions ended. Pre-pandemic lifestyle and medical conditions were derived from previous study center visits.

**Results:**

Among 1850 survey participants (73–98 years; net-response 89%), 74% were at increased risk for severe COVID-19 according to medical conditions; four participants reported SARS-CoV-2 infection (0.2%). Participants reported changes in behavior: 29% refrained from medical appointments, 14% increased TV consumption, 26% reported less physical activity, but no systematic increase of smoking or alcohol consumption. When comparing during- and pre-lockdown reports of lifestyle within participant, we found the same pattern as for the reported perceived changes. Women and the more educated were more susceptible to changes. Worse QOL was perceived by 38%.

**Conclusions:**

Our data suggest that the spring 2020 lockdown did not affect the lifestyle of a majority of the mobile old aged individuals, but the substantial proportions with decreased physical activity and healthcare-seeking are markers of collateral damage.

**Supplementary Information:**

The online version contains supplementary material available at 10.1186/s12877-021-02677-x.

## Introduction

Social life changed drastically in spring 2020 due to the COVID-19 related lockdown in Germany – as in most countries worldwide. While the self-isolation of individuals at high risk, particularly the old aged, was a conceivable option as long as vaccination was unavailable, inflicted lifestyle changes towards a less healthy behavior are important collaterals that warrant attention [1]. Detecting particularly susceptible subgroups can help identify targets for tailored preventive measures [2].

Numerous published studies have highlighted changes in physical activity, smoking, and alcohol consumption. There are many reports on changes towards a less-healthy lifestyle, but also reports on increasing attempts on quitting smoking or drinking [3] or increased exercise on younger adults [4]. However, most published studies included only few or no participants older than 70 or 80 years of age. It is not fully clear to what extent existing results in general adults apply for the old and very old aged. Changes towards a less-healthy lifestyle or reduced healthcare seeking can be particularly critical for old individuals. Therefore, there is a need for study data in the old aged to understand this group’s changes of health-relevant behavior during lockdown.

The limited representation of the old aged in existing studies on lifestyle changes during lockdown is paradox, as the old aged were the most at risk for severe COVID-19 and the most targeted by shielding recommendations before vaccination became available. Changes in behavior need to be put into perspective of the extent of SARS-CoV-2 infections, which were life-threatening in old aged, as well as the extent of pre-existing medical conditions, as these further increased the risk for severe COVID-19 [5]. Between March and July 2020, SARS-CoV-2 has infected more than 35 million individuals and lead to over 1 million confirmed deaths worldwide [6]. Most deaths were in individuals with 70+ years of age. For example in Germany, the individuals aged 70+ constituted 16% of the population, but 86% of COVID-19 related deaths during the first SARS-CoV-2 wave in spring 2020 between March and July 2020 [7].

We thus aimed to investigate the change in health-related lifestyle and healthcare-seeking in old aged individuals in the spring 2020 lockdown in Germany and to relate this to socio-demographic factors, the extent of infection and pre-existing medical conditions in this group. For this, we conducted a follow-up survey of the pre-pandemic well-characterized participants of our AugUR study cohort, a population-based study of individuals aged 70+ living in/near Regensburg, Bavaria [8]. In Bavaria, containment measures were in effect between March 16th to June 16th, 2020; the strict curfew ended in May, 2020. Our questionnaire, mailed by May 12th, 2020, to contactable 2088 AugUR study participants, allowed us to receive timely data on current lifestyle and changes during the first wave of pandemic-related restrictions. Our pre-pandemic information obtained from previous study center visits with interview and medical exams also enabled us to compare during-lockdown reports on lifestyle with pre-lockdown reports and the characterization of pre-existing medical conditions, which had to rely not only on self-reports. There were no study center visits during lockdown in this study as in most other studies.

We here report on the 1850 AugUR study participants, who responded to the AugUR COVID-19 spring 2020 follow-up survey, resulting in a very high response of almost 90%. Specifically, we addressed the following questions:

1. Among AugUR participants, all aged 70+, how many had medical conditions that put these individuals at increased risk for severe COVID-19 irrespective of old age,
2. How many were infected during the first wave and what were their symptoms,
3. What was the extent of self-isolation with regard to use of public transport or doing errands themselves? Did individuals refrain from medical consultations?
4. Compared to before the pandemic/lockdown, did individuals perceive a change in their sedentary behavior, smoking, drinking, or quality-of-life (QOL)? Did the perceived change relate to a change quantified by during- versus pre-pandemic reports?
5. Could we identify subgroups with regard to socio-demographic factors or pre-existing medical conditions that were particularly susceptible to change?

## Subjects and methods

### Study design of the AugUR COVID-19 spring 2020 survey

This survey is a follow-up of our AugUR study participants by a mailed questionnaire. The AugUR study is a population-based cohort study of individuals aged 70+ established before the pandemic in Regensburg, Germany, with the aim to investigate influences on health in the old and very old aged [8]. For all participants of this survey, we had information on medical, socio-demographic, and life style factors from at least one pre-pandemic study center visit.

### AugUR cohort study: population, recruitment, and study program at baseline

The AugUR cohort participants were individuals aged 70+ living in/around Regensburg identified via population registry and recruited in 2013-2019. The study region, city and selected counties of Regensburg, captures ~ 347,000 inhabitants of mostly European ancestry, including 45,000 aged 70+ [8]. A first baseline survey (AugUR-1, 2013-2015) included 1133 participants [8, 9]. A second independent baseline survey (AugUR-2, 2017-2019) included 1316 participants. The study was designed in parallel to the NAKO study to enable cross-comparisons with this Germany-wide cohort recruiting adults 20-69 years oldy. Details on study population, recruitment and study program has been reported previously [8]. Briefly, a random sample of individuals aged 70+ from Regensburg and selected surrounding counties was selected via population registry and invited for the baseline study center visit. In AugUR-2, senior home residents were excluded, as AugUR-1 response in this group was limited. The study program was conducted in the study center at the University Medical Center Regensburg; it included a standardized in-person interview, medical exams, and blood draw by trained staff. As highlighted previously [9, 10], baseline net response was 20%. Since study participants were required to come to the study center, to conduct a 3-h study program, and to answer personally all questions in the interview, we consider our participants as the physically mobile and mentally healthy old aged.

The AugUR study was approved by the Ethics Committee of the University of Regensburg, Germany (vote 12-101-0258). The study complies with the 1964 Helsinki declaration and its later amendments. All participants provided informed written consent.

### Follow-up in the study center before the pandemic

A 3-year follow-up was conducted for AugUR-1 (2016-2018, *n* = 788) and a 6-year follow-up (November 2019 until March 16th, 2020, paused due to the pandemic (*n* = 103). At each follow-up visit, the same study program was conducted as at baseline. A 3-year follow-up for AugUR-2 was planned for May 2020, but postponed due to the pandemic.

### The AugUR COVID-19 spring 2020 survey and questionnaire

**Fig. 1:**
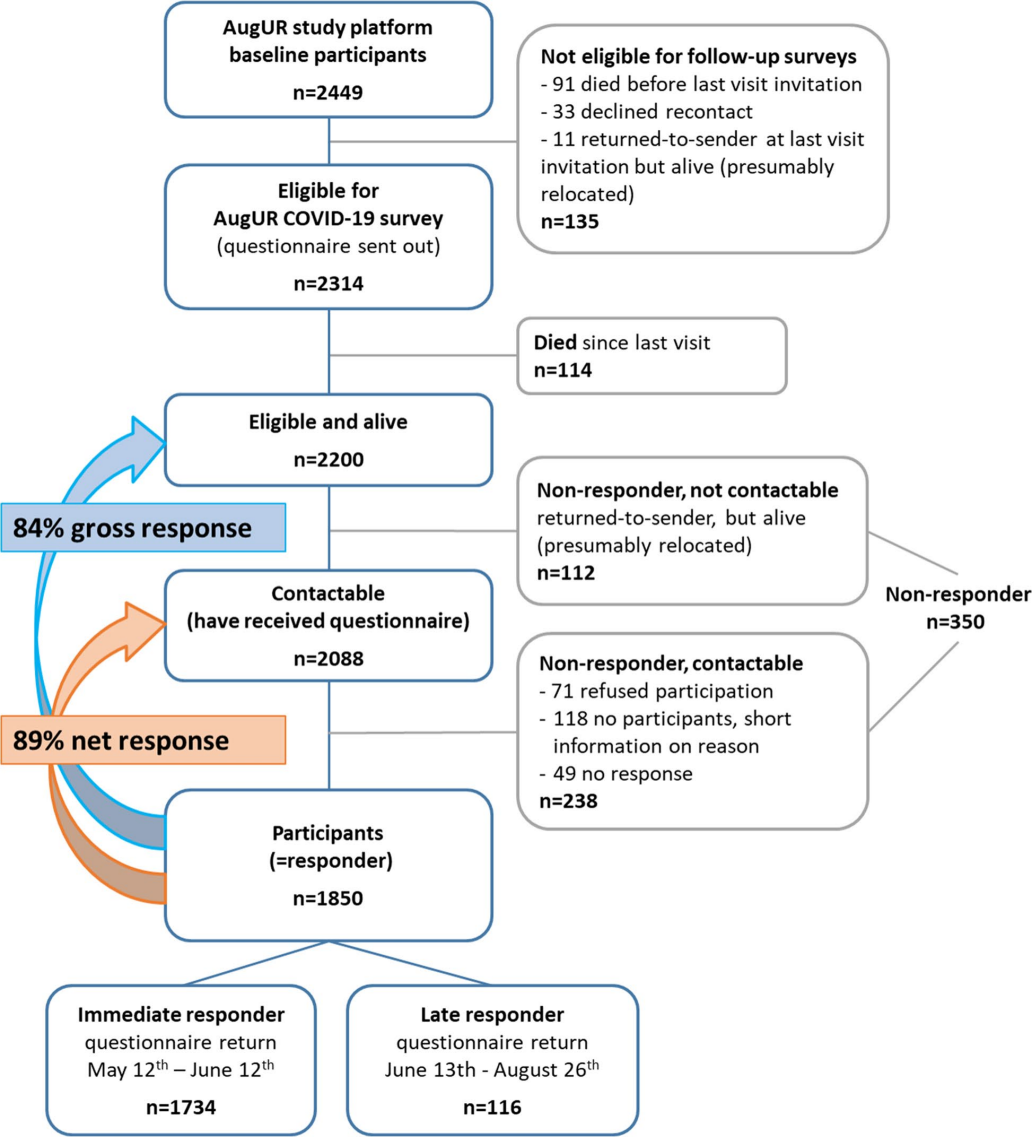
Flow chart of participant inclusion and response. The AugUR study platform initially included 2449 individuals aged 70+, all with informed consent to participate in AugUR research investigations. The questionnaire was sent out to all, for whom we had no adverse information on survival, contactability, or willingness to participate on May 12th, 2020 (*n* = 2314), but contactable (i.e. still residing at the noted address and alive) were only 2088. Of these, 89% have answered to the questionnaire until Aug 26th, 2020 (*n* = 1850)

For the AugUR COVID-19 spring 2020 survey reported in this manuscript, we resorted to the complete AugUR sample of initially 2449 participants, excluding the following: reported dead, reported too ill, not contactable by mail or phone during the recruiting for the prior study center visit, or retracted consent for re-contact (Fig. 1). This rendered 2314 individuals eligible.

A written self-completion questionnaire was sent out per mail to the 2314 eligible individuals on May 11th/12th, 2020. This was shortly after the lift of the curfew in Bavaria (May 6th), but well within the period of contact restrictions (until June 16th). We collected returned questionnaires until August 26th, 2020. The questionnaire was mainly a shortened version of the questionnaire developed by NAKO study investigators and aspects of this questionnaire were also implemented in the Tirschenreuth Study to enable cross-comparisons [11]. Details are given in the Supplementary Note; the full English translation of the questionnaire (originally in German) can be found in the Additional file 2. In brief, we asked about current behavior (as per questionnaire completion) and perceived change since the start of the pandemic (as per Feb 1st, 2020) with regard to: use of public transport, doing errands, healthcare seeking, lifestyle (physical activity, TV consumption, smoking, alcohol consumption), and quality-of-life (QOL). Further questions were on SARS-CoV-2 testing and positive tests, COVID-19 related symptoms, and household.

### Measures to minimize and understand non-response

Individuals returning the questionnaire until June 12th, 2020, were considered immediate responders (with a more immediate impression of the lockdown), those returning it June 12th - Aug 26th as late responders. For all individuals without questionnaire return until July 12th, we implemented measures to minimize and/or understand non-response: (i) we obtained survival status by population registry; (ii) we attempted phone contact and, if successful, reminded individuals to return the questionnaire or conducted the survey as phone interview (late responder); (iii) if we had phone contact, but did not obtain questionnaire information, we asked for the reason of non-response (no interest, no time, too ill, refused to give any information). We divided non-responders (i.e. presumably alive, but did not return questionnaire) into those who did not receive the questionnaire (“returned-to-sender”, i.e. not contactable) and those for whom we had no adverse information that they received the questionnaire (contactable).

### Assessment of socio-demographic factors from baseline visit

From the baseline visit, we obtained year of birth, sex, and years of education (from type/duration of schools visited/finished, formal training via vocational college/universities).

### Assessment of medical conditions and pre-pandemic lifestyle at prior visit

For all participants of the AugUR COVID-19 spring 2020 survey, we derived information on medical conditions and lifestyle from their prior study center visit (April 2016 - March 16th, 2020), which had included a face-to-face interview, medical exams and bio-probing [8, 9].

Body mass index (BMI) was computed based on measured weight in light clothing and measured height, obesity defined as BMI ≥ 30 kg/m^2^, HbA1c and serum creatinine measured in fresh blood, and chronic kidney disease defined as estimated glomerular filtration rate (eGFR) < 60 mg/dl/1.73m^2^ [12, 13]. Further medical history was assessed via self-report: cancer (excluding white skin cancer), type 2 diabetes mellitus, hypertension, chronic bronchitis, asthma, serious heart conditions (i.e. history of myocardial infarction OR percutaneous coronary intervention OR coronary bypass surgery OR heart weakness) and cerebrovascular disease (i.e. history of stroke). The Center for Disease Control and Prevention (CDC) classifies individuals of any age at increased risk for severe COVID-19 (i.e. hospitalization, intubation/ventilation, or death) based on medical conditions, from which we extracted conditions common in the elderly: cancer, chronic kidney disease, chronic bronchitis, obesity, serious heart conditions, or type 2 diabetes [5]. CDC lists further conditions as possible risk factors for severe COVID-19, from which we extracted asthma, hypertension, cerebrovascular disease, current/former smoking [5].

Pre-pandemic reports on lifestyle included number of cigarettes smoked daily, number of alcoholic drinks consumed daily, and physical activity categories as well as QOL (on a scale from 0, worst, to 100, best). For this work, we derived this information from the latest study center visit most closely before the start of the lockdown in March 2020. The pre-pandemic information on lifestyle was assessed with the same questions as in the above-mentioned COVID-19 instrument, but different data collection method (standardized face-to-face interview via trained staff instead of a self-administered questionnaire) [8, 9].

### Data management and statistical analyses

Data management and statistical analyses were conducted via SAS 9.4 software (SAS Institute Inc., Cary, NC, USA) and IBM SPSS Statistics for Windows, Version 26.0 (IBM Corp., Armonk, NY, USA). Questionnaire responses were summarized using percentages for categorical variables and mean and standard deviation or median an interquartile range (IQR) for continuous variables. Perceived changes in behavior were described as three categories (same, less, more than before). Sensitivity analyses were conducted restricting to immediate responders (i.e. questionnaire return by June 12th, 2020), who were still under the direct impression of the contact restrictions when filling out the questionnaire.

Changes in lifestyle factors as well as QOL were also quantified using reports for current behavior of the COVID-19 spring 2020 survey with reports at the prior study center visit (change in physical activity category, difference in reported number of cigarettes smoked daily, difference in reported number of alcoholic drinks consumed daily, difference in QOL-score). Variation in the continuous change variables were visualized by perceived change category. Sensitivity analyses were conducted restricting to the individuals with a more recent prior study center visit (i.e. < 12 months before lockdown, i.e. March 2019 – March 2020, balancing seasonal variation) to separate changes more likely from lockdown rather than time elapsed and respective aging.

To identify susceptible subgroups, we analysed the association of socio-demographic factors (age, sex, education, living alone) and pre-existing medical conditions (i.e. being at increased risk for severe COVID-19 independent of old age according to CDC [5]) with changes in behavior by multivariable linear/logistic regression. Statistical significance was judged at 5%. We also evaluated age-by-sex and sex-by-education interaction, resorting to the model without interaction when the interaction term was not statistically significant.

## Results

### Net response of the AugUR COVID-19 spring 2020 survey was very high

Among the 2314 individuals to whom the questionnaire of the AugUR COVID-19 spring 2020 survey was sent out, 2088 individuals were contactable (i.e. alive and received questionnaire) and the filled-out questionnaire was returned by 1850 individuals (“AugUR COVID-19 survey participants”, questionnaire completion May 13th to Aug 26th, 2020). This resulted in a net response of 89% (Fig. 1), lower among women than men (87% versus 90%) and among the very old (80+) versus 73-79 years (91% versus 87%). The 1850 participants included 48% men, survey age ranged from 73 to 98 years (birth years 1922 – 1947), 5% were smoker and mean BMI was 27.6 kg/m^2^ (Table 1). Few women, but 57% of men had ≥13 years of education. One main reason for non-response was illness (39% too ill among 110 in non-responder phone follow-up). When comparing the lost-to-follow-up (350 non-responders, 114 died since prior visit) with the 1850 participants based on information from the prior study center visit, we found fewer men, more smoker, lower QOL, and less physical activity (Table 1).

**Table 1:** Characteristics of participants and those lost to follow-up. Characteristics of all AugUR study cohort participants were derived from information assessed at the prior study center visit (before lockdown, March 2020) from face-to-face interview, medical exams, and serum measurements (*n* = 2314). Shown are characteristics for (i) 1850 participants of this AugUR COVID-19 survey, (ii) 1734 participants with immediate response (i.e. questionnaire return May 12th – June 12th, 2020), (iii) 524 among the 1850 participants seen within 1 year before lockdown (i.e. March 2019 – March 2020), (iv) 350 non-responders (known to be alive, not participating in this survey, consent to be part of AugUR study platform; 112 not contactable, 238 contactable), (v) 114 who died between prior visit and this survey. Shown is median [inter-quartile range], if not noted otherwise

Participant characteristics	Participants ***n*** = 1850	Participants immediate response ***n*** = 1734	Participants seen within 1 year before lockdown ***n*** = 524	Non-responders ***n*** = 350	Died ***n*** = 114
Year of birth	1922 - 1947	1922 - 1947	1924 - 1947	1921 - 1947	1919 - 1946
Age [yrs] at prior visit, median (min-max)	78.8 (70-96)	78.7 (70-96)	79.5 (72-95)	80.7 (71-97)	83.8 (71-98)
Age [yrs] at survey/death, median (min-max)	80.5 (73-98)	80.4 (70-96)	80.1 (73-96)	82.9 (71-97)	84.7 (71-98)
Men, % (n)	47.5 (878)	48.0 (833)	46.2 (242)	40.0 (140)	61.4 (70)
Years of education^a^	11 [10 - 14]	11 [10-15]	11 [10-15]	10 [10 - 13]	11 [10 - 13.3]
Quality of life^b^	75 [60 - 85]	75 [60 - 85]	75 [60-85]	70 [50 - 80]	62.5 [50 - 80]
Physically active^c^, % (n)	80.6 (1478)	81.1 (1395)	78.4 (407)	68.3 (235)	56.9 (62)
Current smoker/Ex-smoker^d^, % (n)	4.8 (88) / 38.1 (703)	4.7 (82) / 38.2 (660)	3.6 (19) / 37.6 (196)	4.9 (17) / 37.8 (130)	6.1 (7) / 39.5 (45)
# cigarettes smoked daily^e^	6.0 [4.0 - 15.0]	6.0 [4.0 - 15.0]	5.0 [4.3-10.0]	10.0 [4.3 - 27.5]	12.5 [5 - 18.5]
# alcoholic drinks daily^f^,	0.54 [0.15 - 1.18]	0.54 [0.15 - 1.18]	0.54 [0.15-1.18]	0.54 [0.15 - 1.18]	0.54 [0.15 - 1.18]
eGFR [mg/dl/1.73m^2^], mean ± SD	68.4 ± 16.0	68.4 ± 15.9	67.3 ± 16.9	65.0 ± 17.3	61.8 ± 22.9
HbA1c [%], mean ± SD	5.78 ± 0.65	5.78 ± 0.65	5.68 ± 0.59	5.84 ± 0.75	5.97 ± 0.81
Body-mass-Index^f^ [kg/m^2^]^g^, mean ± SD	27.6 ± 4.4	27.6 ± 4.4	27.6 ± 4.2	28.4 ± 4.9	26.7 ± 5.3

*SD* standard deviation, *IQR* interquartile range, *eGFRcrea* estimated glomerular filtration rate based on serum creatinine measurements

^a^School education from 6 years (no final exam) to 13 years (high school graduation), professional/university training from 0 years (none) to 11 years (professional training, university, doctoral thesis). ^b^Scale from 0 (very poor) to 100 (excellent). ^c^Light regular activity (includes bicycling, gardening, walking) in summer and/or winter, weekly for > 2 h (active), or less (not active). ^d^Current smoker (as per prior visit), *n* = 88; ex-smoker having stopped smoking for ≥1 month, *n* = 703. ^e^Currently smoking (as per prior visit), *n* = 73 with information on #cigarettes. ^f^For individuals with any alcohol consumption during the last 12 months (as per prior visit), computed as reported frequency of drinking times the number of drinks (one drink defined as a small bottle of beer, 0,33 l, a small glass of wine, 0,125 l, or liquor, 4 cl.); *n* = 1716 with information on #drinks. ^g^Measured weight in kg divided by squared measured body height in m

### Majority of participants was at increased risk for severe COVID-19 by medical conditions

**Fig. 2:**
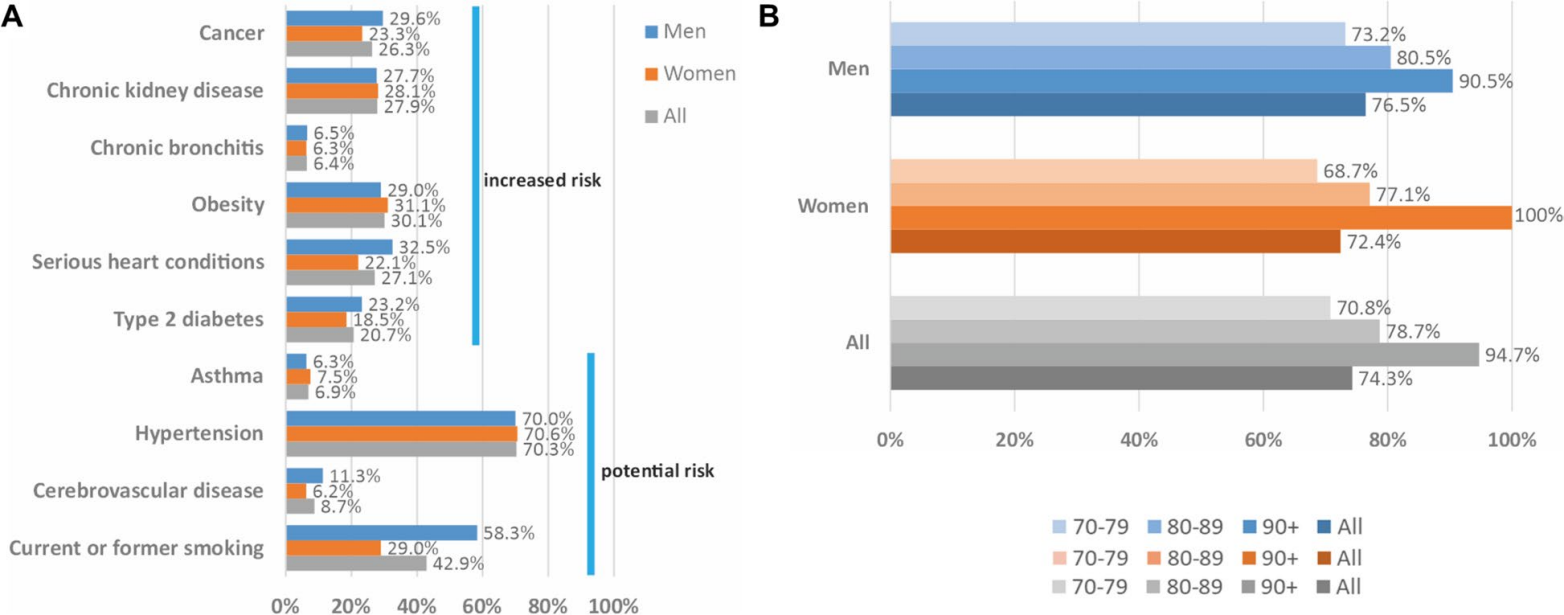
Frequency of participants at increased risk for severe COVID-19 beyond old age. Shown are frequencies of individuals with medical conditions assessed at the prior study center visit (among the 1850 participants of this survey): **A** having a medical condition listed to increase or possibly increase risk for severe COVID-19 [5], **B** having ≥1 condition listed to increase risk (cancer, chronic kidney disease, chronic bronchitis, obesity, serious heart conditions, type 2 diabetes) [5] by 10-year age-groups and sex (blue and orange), men&women combined by age-group and all combined (gray)

For individuals at any age, the CDC classifies medical conditions with strong evidence for increased risk for severe COVID-19 [5], most of which are common in the elderly (cancer, chronic kidney disease, chronic bronchitis, obesity, serious heart conditions, or type 2 diabetes). Based on the information assessed at the prior study center visit (mean time between survey and prior visit = 1.8 years; < 1 year: *n* = 524, 28%; 1-3 years: *n* = 1029, 56%; > 3 years: *n* = 297, 16%), we derived frequencies of these medical conditions (Fig. 2A, Supplementary Table 1). We found 74% of our 1850 participants with at least one of these conditions and thus at increased risk for severe COVID-19 (Fig. 2B). This risk group was larger among men than women (76% versus 72%), mostly due to more men with serious heart conditions, and the risk group increased by 10-year age-group (71, 79, 95% for those aged 70-79, 80-89, 90+, respectively; Fig. 2B). When extending to CDC conditions listed as possible risk factors for severe COVID-19 [5] (asthma, hypertension, cerebrovascular disease, current/former smoking), the risk group increased to 94% among the 1850 participants (Supplementary Table 1). In summary, the majority of participants was at increased risk for severe COVID-19 beyond old age by pre-existing medical conditions.

### Only four participants reported infection and all had mild consequences

We asked whether participants had undergone testing for SARS-CoV-2 infection and whether any test result had been positive. Among the 1850 participants, 52 reported a test (test dates March 21th - June 15th, 2020; reasons for testing: contact to infected, symptoms, returning from risk areas, other, *n* = 5, 15, 0, or 19, respectively). Four were tested positive (8% of 52, 0.2% of 1850): their age ranged from 76 to 95 years, three men, all non-smoking, three at increased risk for severe COVID-19 due to medical conditions. Two reported to live alone, two with partners; the partners were also tested, but not infected. Their QOL ranged from 50 to 80 (IQR of all at survey 50-80, at prior visit 60-85 on a scale 0, worst, to 100, best).

All participants, irrespective of infection or previous SARS-CoV-2 testing, were asked about experienced symptoms since Feb 1st. 2020. Of the 1850 participants, 23% reported at least one symptom considered COVID-19 related (cough, shortness of breath, respiratory problems, fever/chills, or loss of taste/smell [14], Supplementary Table 2). A loss of taste or smell, considered specific to SARS-CoV-2 infection, was reported by 2% (none of the four infected). Two of the four infected reported any symptom (cough, difficulty breathing, pain in extremities, diarrhea, headache, rhinitis), but none of the four reported bronchitis or pneumonia.

**Fig. 3:**
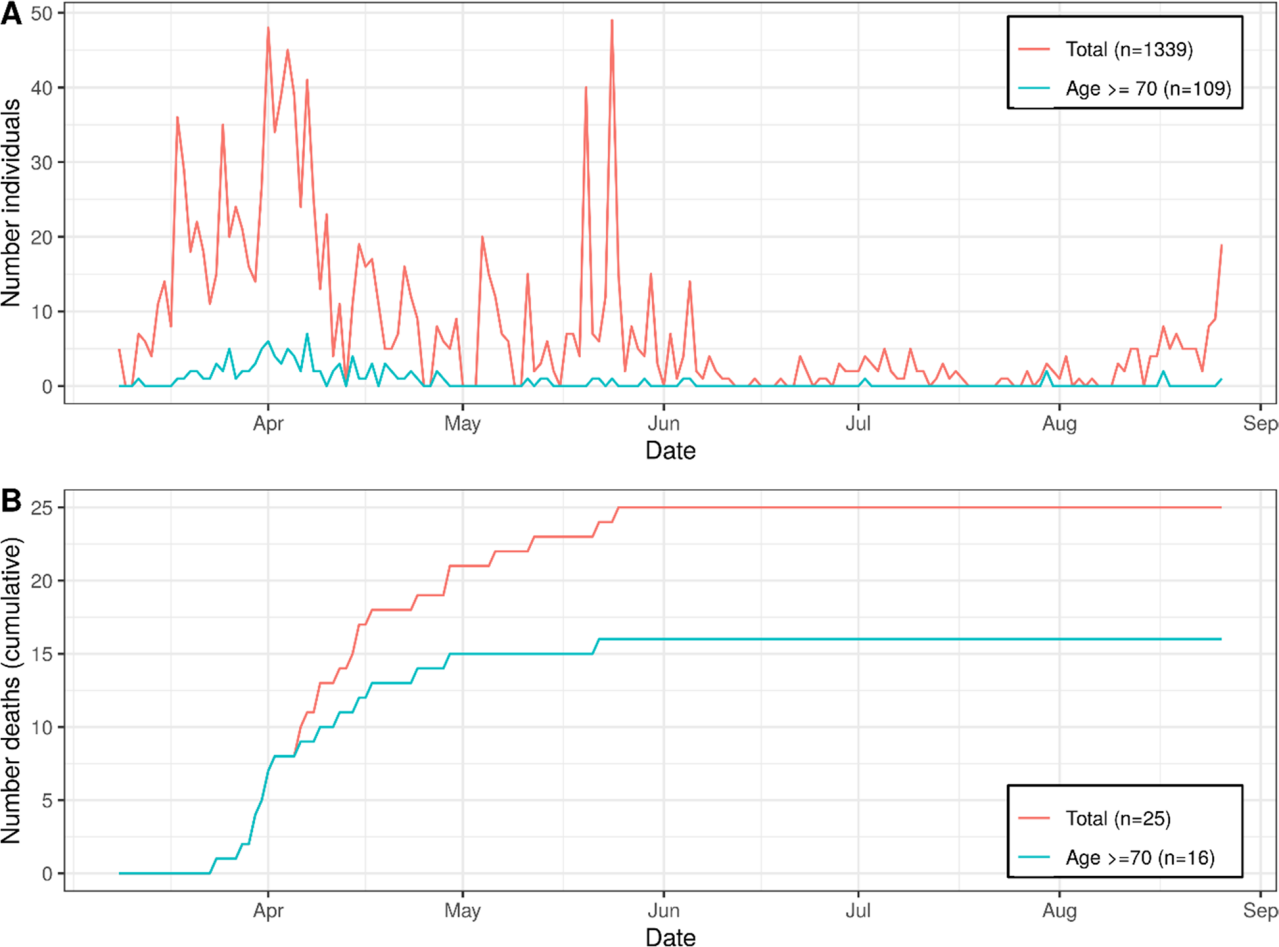
The SARS-Cov-2 epidemic situation in the study capture area until August 2020 for all inhabitants and those aged 70+. We derived the numbers of SARS-CoV-2 infections and COVID-19 related deaths in the study area (city and county of Regensburg) from the Bavarian Food and Health Safety Authority (Landesamt für Gesundheit und Lebensmittelsicherheit) for the survey observation period (Feb 1st to Aug 26th, 2020). Shown are (**A**) number of newly reported infected per day, (**B**) cumulative number of deaths. Those aged 70+ comprise 13% of study region residents, 8% of infected, and 64% of COVID-19 related deaths

When linking these observations to the infection occurrence among the 46,461 inhabitants aged 70+ in the study capture area (infections mostly March – June 2020, Fig. 3A), we found the proportion of positive tested (0.3%; *n* = 109) and the 4.3 expected individuals with infection among the 1850 participants to fit well to our observation. Given the 16 individuals aged 70+ in the study area who died with COVID-19 (0.03% of the 46,461), the expected number of 0.6 deaths among the 2314 eligible individuals indicated little to no bias from COVID-19 related death. Of note, those aged 70+ comprised 13% of study region inhabitants, 8% of those tested positive, and 64% of those with COVID-19 related death (Fig. 3B).

### Household and some aspects of outside contacts during first wave lockdown

We were interested in how the number of infected participants, which were very few, related to participants’ isolation during the first wave lockdown (March 2020 to June 17th, 2020), when old aged individuals were advised to avoid public transportation and doing errands themselves. Larger households, particularly when including younger household members, were reported to increase risk of infection [15]. Of the 1850 participants, 36% reported to live alone (more women than men), 62% lived with at least one more person in a private household and 1% in a senior residence (Table 2). At the time of questionnaire completion (May 13th to Aug 26th, 2020), 92% reported at least one of the following: 81% of participants reported to do their own errands, 26% to use public transportation, 18% had a help come to their home, 3% lived with a younger generation person in the household, and 1% had contact with an infected person (Table 2). Since the lockdown ended June 16th, 2020, we conducted sensitivity analyses restricting to the 1734 participants with immediate response (i.e. questionnaire return until June 12th, 2020). This yielded the same results (Supplementary Table 3). Overall, most participants sustained at least some type of outside contacts.

**Table 2:** Household and outside contacts during first wave lockdown among 1850 participants. Shown is the household situation and some aspects of behavior involving outside contacts for the 1850 participants (via self-completion questionnaire). Sensitivity analyses restricting to immediate responders (May 13th to June 12th, 2020) showed the same (Supplementary Table 3)

Household and outside contacts	Overall ***n*** = 1850	Women ***n*** = 972	Men ***n*** = 878
**Household**			
Living alone, % (n)	36.4 (664)	50.9 (489)	20.3 (175)
Living with **≥**1 person, % (n)	62.3 (1137)	47.7 (458)	78.7 (679)
Living in a nursing home, % (n)	1.3 (23)	1.5 (14)	1.0 (9)
**Outside contacts**			
Contact with infected person^b^, % (n)	1.0 (18)	0.8 (7)	1.3 (11)
Living with younger generation^a^, % (n)	2.7 (50)	2.0 (19)	3.5 (31)
Using public transport^c^, % (n)	25.6 (465)	30.5 (291)	20.2 (174)
Doing errands^c^, % (n)	81.4 (1488)	80.8 (778)	82.1 (710)
Having a help come to the household^c^, % (n)	18.0 (325)	19.7 (186)	16.1 (139)

^a^Defined as additional person in household with < 50 years of age. ^b^Contact for > 15 min at a distance < 1.5 m or person in the same household infected. ^c^Defined as ever using public transport / ever doing errands / ever having a help come to the household during February 1st until July 12th, 2020

Available n (overall, women, men): Contact with infected person 1783/ 927 / 856; living with younger generation person 1824/ 961 / 863; using public transport 1814/ 953 / 861; doing errands 1828/ 963 / 865; having a help come to the household 1809/ 945 / 864;

### Participants reduced outside contacts and refrained from medical appointments

We asked participants whether they had changed their behavior with regard to public transport, obtaining food, or healthcare seeking at the time of questionnaire completion compared to before the pandemic (as of Feb 1st, 2020). A substantial proportion reported less use of public transport, less errands on their own, and increased food delivery (33, 42, 29%, respectively, Table 3), which was more pronounced among women than men; rather few reported the opposite change.

**Table 3:** Perceived changes in behavior and QOL among 1850 participants. Shown are perceived changes reported at questionnaire completion (questionnaire return May 13th to Aug 26th, 2020) to before Feb 1st, 2020. Sensitivity analyses restricting to the 1734 immediate responders (return until June 12th, 2020) showed the same (Supplementary Table 4)

Perceived changes (now vs. before pandemic)	Overall^**a**^ ***n*** = 1850	Women ***n*** = 972	Men ***n*** = 878	Age 73-79 at survey ***n*** = 829	Age 80+ at survey ***n*** = 1021
**Refraining from medical cons.**					
No % (n)	71.1 (1213)	68.0 (598)	74.5 (615)	71.7 (562)	70.7 (651)
Yes, % (n)	28.9 (492)	32.0 (282)	25.5 (210)	28.3 (222)	29.3 (270)
Rescheduling, % (n)	21.5 (367)	23.1 (203)	19.9 (164)	22.4 (176)	20.7 (191)
Despite acute need, % (n)	0.9 (16)	1.1 (10)	0.7 (6)	0.9 (7)	1.0 (9)
Despite regular, % (n)	6.4 (109)	7.8 (69)	4.8 (40)	5.0 (39)	7.6 (70)
**Using public transport**					
Less, % (n)	555 (33.1)	350 (40.1)	205 (25.5)	246 (32.0)	309 (34.0)
Same, % (n)	1068 (63.7)	487 (55.8)	581 (72.2)	501 (65.1)	567 (62.4)
More, % (n)	54 (3.2)	35 (4.0)	19 (2.4)	22 (2.9)	32 (3.5)
**Doing errands**					
Less, % (n)	754 (41.8)	473 (49.9)	281 (32.9)	344 (42.6)	410 (41.2)
Same, % (n)	1025 (56.8)	466 (49.2)	559 (65.4)	451 (55.8)	574 (57.7)
More, % (n)	24 (1.3)	9 (0.9)	15 (1.8)	13 (1.6)	11 (1.1)
**Getting food delivered**					
Less, % (n)	9 (0.5)	6 (0.7)	3 (0.4)	3 (0.4)	6 (0.7)
Same, % (n)	1190 (70.7)	569 (66.1)	621 (75.5)	559 (72.8)	631 (68.9)
More, % (n)	485 (28.8)	286 (33.2)	199 (24.2)	206 (26.8)	279 (30.5)
**Physical activity**^**b**^					
Less, % (n)	25.8 (456)	30.5 (281)	20.7 (175)	25.0 (200)	26.5 (256)
Same, % (n)	72.1 (1273)	67.4 (620)	77.2 (653)	72.3 (579)	71.9 (694)
More, % (n)	2.1 (37)	2.1 (19)	2.1 (18)	2.7 (22)	1.6 (15)
**TV consumption**					
More, % (n)	14.0 (259)	18.0 (169)	10.6 (90)	16.0 (129)	13.3 (130)
Same, % (n)	81.0 (1498)	80.6 (755)	87.3 (743)	82.5 (666)	84.8 (832)
Less, % (n)	1.7 (31)	1.4 (13)	2.1 (18)	1.5 (12)	1.9 (19)
**Smoking**^**c**^					
More, % (n)	7.4 (4)	10.7 (3)	3.8 (1)	11.8 (4)	0.0 (0)
Same, % (n)	81.5 (44)	78.6 (22)	84.6 (22)	73.5 (25)	95.0 (19)
Less, % (n)	11.1 (6)	10.7 (3)	11.5 (3)	14.7 (5)	5.0 (1)
**Alcohol consumption**^**d**^					
More, % (n)	2.3 (39)	2.3 (16)	3.1 (23)	2.9 (19)	2.6 (20)
Same, % (n)	94.8 (1350)	95.9 (661)	93.7 (689)	93.8 (618)	95.7 (732)
Less, % (n)	2.0 (35)	1.7 (12)	3.1 (23)	3.3 (22)	1.7 (13)
**Perceived QOL**					
Worse, % (n)	38.3 (668)	40.7 (370)	35.6 (298)	39.7 (316)	37.1 (352)
Same, % (n)	61.4 (1072)	59.0 (536)	64.1 (536)	60.1 (478)	62.6 (594)
Better, % (n)	0.3 (5)	0.3 (3)	0.2 (2)	0.3 (2)	0.3 (3)

^a^total n is slightly different for each variable (*n* = sum of the respective rows to compute proportions). ^b^Including bicycling, gardening, walking. ^c^Among current smoker as per survey (*n* = 54), defined as currently smoking ≥1 cigarette per day. ^d^For individuals with any alcohol consumption during the last 12 months (as per survey, *n* = 1424)

Almost a third (29%) refrained from medical consultations, more women than men, but no difference when comparing the 80+ to the 73- to 79-year-old (Table 3). When restricting to the 1734 immediate responders, we found the same (Supplementary Table 4). Decreased healthcare-seeking behavior can be potentially problematic. We were thus interested whether we could identify susceptible subgroups. When analyzing the association of socio-demographic factors (age, sex, education, living alone) and pre-existing medical conditions with the odds of having refrained from medical consultation by logistic regression, this indicated higher susceptibility in women (OR = 1/0.7 = 1.4 across models, *P* < 0.003, Supplementary Table 5A). Notably, we do not know how many medical appointments were canceled by physicians or hospitals.

### Participants reported a change towards less physical activity, but not for increased smoking or alcohol consumption

We asked participants whether they had changed their lifestyle with regard to sedentary or addictive behavior (physical activity, TV consumption, smoking, and alcohol consumption) at the time of questionnaire completion compared to before Feb 1st, 2020 (same, less now, more now). A quarter (26%) reported that they were less physically active versus 2% more active and 14% with more TV consumption versus 2% less, both more pronounced in women (Table 3). There was no trend towards more smoking or more alcohol consumption (7% more smoking vs. 11% less, 2% with more alcohol consumption vs. 2% with less; Table 3). The majority of participants, 60% (52% among women, 68% among men), did not report any change in these lifestyle factors. Sensitivity analyses restricting to the 1734 immediate responders yielded similar results (Supplementary Table 4). We were interested to identify subgroups particularly susceptible to change. When modelling the association of socio-demographic factors and pre-existing medical conditions with the odds of perceived decreased physical activity or increased TV consumption by logistic regression, we found significantly increased odds for increased TV consumption among the old aged versus the very old (73-79 years vs. 80+), among women, higher educated, and those living alone (*P* < 0.01, Supplementary Table 5A). For decreased physical activity, higher odds were found for women (OR = 1/1.7 across models, *P* < 0.001) and a tendency for the higher educated (Supplementary Table 5A).

### Lifestyle changes quantified from during and pre-lockdown information showed a similar pattern as perceived changes

While the report that one’s own lifestyle was perceived as having changed is a noticeable parameter, we also derived the “quantified” change by comparing the current (i.e. during lockdown) with the pre-pandemic report of the respective factor. First, we aimed to understand dependencies of during-lockdown reports of lifestyle factors (physical activity category, cigarettes smoked daily, alcoholic drinks consumed daily) on socio-demographic factors (age, sex, education, living alone) and pre-existing medical conditions. We found all evaluated factors, but not “living alone”, to be associated with at least one lifestyle factor (Supplementary Table 5B).

Second, we quantified the difference between during- and pre-lockdown reports for each of the 1850 participants (change in physical activity category, difference in number of cigarettes, difference in number of drinks) and analyzed also the sample restricted to the 524 individuals with prior visit < 1 year before lockdown (March 2019- March 2020). We found 24% with decreased versus 5% with increased physical activity (19% versus 8% among the 524), almost no difference in cigarettes smoked and no difference in drinks consumed among the 1850 participants and in the 524 sub-set (Table 4).

**Table 4:** Lifestyle and QOL reported at survey, at prior visit, and quantified difference among the 1850 participants. Shown are self-reported lifestyle factors and QOL from the AugUR COVID-19 survey and the prior study center visit, overall (April 2016 – March 2020, mean time between survey and prior visit = 1.76 years, SD = 0.93) and restricted to those seen < 1 year before lockdown (Nov 2019 – March 2020). Reported is median [25th-75th percentile], if not indicated otherwise

	Overall ***n*** = 1850	Women ***n*** = 972	Men ***n*** = 878	Age at prior visit 70-79 ***n*** = 1095	Age at prior visit 80+ ***n*** = 755
**Physical activity**^**a**^					
At survey					
yes, % (n)	61.4 (1118)	56.9 (541)	66.3 (577)	67.5 (730)	52.5 (388)
no, % (n)	38.6 (703)	43.1 (410)	33.7 (293)	32.5 (352)	47.5 (351)
At prior visit, all					
yes, % (n)	80.6 (1478)	79.6 (767)	81.7 (711)	84.4 (920)	75.0 (558)
no, % (n)	19.4 (356)	20.4 (197)	18.3 (159)	15.6 (170)	25.0 (186)
At prior visit, < 1 year before					
yes, % (n)	78.4 (407)	78.5 (219)	78.3 (188)	81.3 (230)	75.0 (177)
no, % (n)	21.6 (112)	21.5 (60)	21.7 (52)	18.7 (53)	25.0 (59)
Difference*, all					
Less, % (n)	24.3 (439)	27.0 (255)	21.3 (184)	21.7 (234)	28.2 (205)
Same, % (n)	70.4 (1272)	68.3 (645)	72.7 (627)	73.5 (792)	65.9 (480)
More, % (n)	5.3 (95)	4.7 (44)	5.9 (51)	4.8 (52)	5.9 (43)
Difference*, < 1 year before					
Less, % (n)	18.6 (95)	21.2 (58)	15.6 (37)	17.5 (49)	20.0 (46)
Same, % (n)	73.7 (376)	70.7 (193)	77.2 (183)	75.7 (212)	71.3 (164)
More, % (n)	7.6 (39)	8.1 (22)	7.2 (17)	6.8 (19)	8.7 (20)
**# cigs smoked daily**^**b**^					
At survey	5.0 [0.0-10.0]	5.0 [0.8-10.0]	5.5 [0.0-16.0]	7.0 [1.0-11.0]	3.0 [0.0-5.6]
At prior visit, all	7.0 [4.8-16.0]	6.0 [4.0-10.5]	10.5 [5.0-20.0]	10.0 [5.0-20.0]	6.0 [3.0-10.0]
At prior visit, < 1 year before	5.0 [4.0-10.0]	5.0 [5.0-10.0]	7.6 [0.0-18.8]	5.0 [4.0-10.0]	NA (*n* = 0)
Difference*, all	(−1.0) [(−6.0)-0.0]	(−0.1) [(−5.0)-0.0]	(−2.0) [(−10.0)-0.0]	(−0.1) [(−6.0)-0.0]	(−2.0) [(−6.5)-0.0]
Difference*, < 1 year before	0 [(− 2.0)-1.0]	0 [(− 2.0)-5]	(− 0.1) [(−3.8)-0.8]	0 [(− 2.0)-1.0]	NA (*n* = 0)
**# alcoholic drinks daily**^**c**^					
At survey	0.6 [0.1-1.5]	0.2 [0.0-0.6]	0.6 [0.1-1.5]	0.6 [0.1-1.5]	0.6 [0.1-1.5]
At prior visit, all	0.5 [0.2-1.2]	0.2 [0.1-0.5]	1.2 [0.5-1.2]	0.5 [0.2-1.2]	0.5 [0.2-1.2]
At prior visit, < 1 year before	0.5 [0.2-1.2]	0.5 [0.2-1.2]	1.2 [0.2-1.2]	0.5 [0.2-1.2]	0.5 [0.2-1.2]
Difference*, all	0.0 [(−0.4)-0.3]	(−0.05) [(− 0.2)-0.1]	0.0 [(− 0.5)-0.3]	0.0 [(− 0.4)-0.3]	0.0 [(− 0.4)-0.3]
Difference*, < 1 year before	0.0 [(− 0.2)-0.3]	0.0 [(− 0.01)-0.1]	0.0 [(− 0.4)-0.3]	0.0 [(−0.1)-0.3]	0.0 [(− 0.4)-0.3]
**QOL score (0-100)**^**d**^					
At survey	70 [50-80]	70 [50-80]	75 [60-85]	75 [60-85]	70 [50-80]
At prior visit, all	75 [60-85]	75 [60-80]	80 [65-85]	80 [70-85]	75 [50-80]
At prior visit, < 1 year before	75 [60-85]	75 [58-80]	80 [65-85]	80 [65-85]	75 [55-80]
Difference*, all	0 [(−15)-10]	0 [(−15)-10]	0 [(−15)-10]	0 [(−15)-10]	0 [(− 15)-10]
Difference*, < 1 year before	0 [(−10)-10]	0 [(− 10)-10]	0 [(−10)-10]	0 [(−10)-10]	0 [(− 10)-10]

Abbreviations: *QOL* quality of life; *Difference survey vs. prior visit. ^a^Physical activity as categories of weekly hours of light activity (0, 0-2 h, > 2 h; including bicycling, gardening, walking). ^b^Among current smokers at prior visit OR current smoker at survey with # cigs reported at prior visit AND at survey (*n* = 66). ^c^Among those who had consumed any alcohol for the last 12 months at prior visit OR at survey with # drinks reported (*n* = 1580); one drink was defined as a small bottle of beer, 0,33 l, a small glass of wine, 0,125 l, or liquor, 4 cl. ^d^Among those who had reported QOL score at prior visit AND at survey (*n* = 1715); scale ranging from 0 (very poor) to 100 (excellent)

Third, we modelled the association of socio-demographic factors and pre-existing medical conditions with quantified change restricting to the 524 sub-set (for physical activity and drinking, not for smoking due to only 14 smokers). We found no significant effect from any of the included covariates, except a reduced number of alcoholic drinks by increased age (*P* < 0.001) and a tendency of reduced physical activity for women (Supplementary Table 5C).

**Fig. 4:**
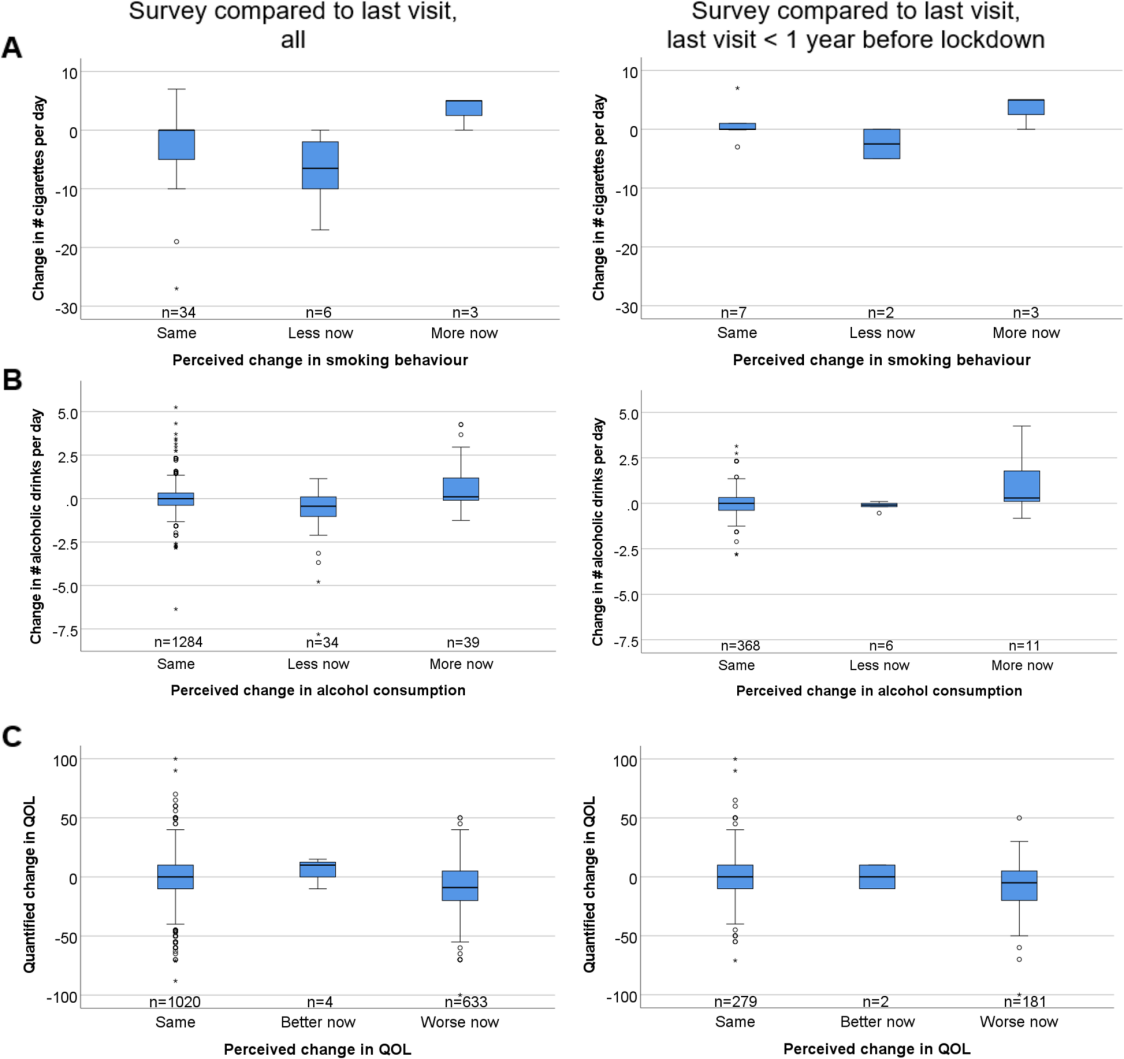
Comparing quantified differences in lifestyle factors and QOL with perceived changes. We derived categories of perceived changes in lifestyle and QOL reported during lockdown (same, less/better now, more/worse now) with the quantified change of the report during lockdown compared to the report pre-lockdown. By category of perceived change, we show the distribution of the quantified change for all participants (prior visit April 2016 – March 2020, *n* = 1850, mean time before lockdown = 1.76 years, SD = 0.93; left column) and restricted to those with prior visit < 1 year before lockdown (March 2019 – March 2020, *n* = 524; right column) where information on both perceived and quantified changes was availble. Shown are (**A**) difference in number of cigarettes smoked daily (among current smokers at survey or prior visit, 43 smokers in left column, 13 smokers in right column), (**B**) difference in number of alcoholic drinks consumed daily (among alcohol consumers at survey or prior visit, *n* = 1357 or 385, respectively), (**C**) difference in QOL score (*n* = 1657 or 462, respectively)

Fourth, when comparing the pattern of quantified change by the categories of perceived change (same, less now, more now), we found a consistent pattern in the 1850 (Fig. 4A-C right column), also when restricting to the 524 individuals with prior visit < 1 year before lockdown (Fig. 4A-C left column). Overall, the evaluation of quantified change supported the findings for perceived change.

### A large proportion of participants perceived a worse QOL

The situation during the lockdown in March 2020 was exceptional and potentially hard on the QOL, particularly for this old age group. We thus asked participants whether they perceived a change in QOL (same, better now, worse now) compared to Feb 1st, 2020. For the 1850 participants, the majority reported no change (61%), but a substantial proportion perceived a change towards worse QOL (38% worse, 0.3% better; Table 3), more pronounced among women than men (41 and 36%, respectively). When modelling socio-demographic factors for association with a perceived worsening of QOL, we found women (*P* < 0.05, all models) and the higher educated (*P* < 0.001, all models) to be more susceptible (Supplementary Table 5A).

While the report of a perceived worsening of QOL is a noteworthy feeling of the participant, we also derived the quantified change by comparing QOL scores reported during lockdown with reports pre-lockdown within participants. We found a median of zero difference in the 1850 participants and in the 524 sub-set with prior visit < 1 year before lockdown with small variation (IQR (− 15) to (+ 10) and (− 10) to (+ 10), respectively; Table 4). When modelling socio-demographic factors for association with quantified difference in QOL restricting the 524, we found no association (Supplementary Table 5C). When comparing the quantified change in QOL by categories of perceived change, we found some consistent pattern of quantified change in QOL with perceived change, which, however, almost disappeared when restricting to the 524 participants with prior visit < 1 year before lockdown (Fig. 4C). Notably, QOL reported at survey was highly associated with pre-existing medical conditions independent of socio-demographic factors: mean QOL among 80-year-old women was 69 points, without and with adjustment for education and living alone (model I, model II, Supplementary Table 5B), but increased to 73 points among 80-year-old women without medical conditions; medical conditions decreased QOL significantly by 5 points (model III, *P* < 0.001). This suggests that the QOL, despite being a rough scale, captured aspects of overall health.

Overall, the large majority of participants reported no change in QOL, but 38% perceived a worse QOL, more women and higher educated, but the reported QOL score during lockdown was surprisingly similar to the QOL score reported pre-lockdown.

## Discussion

We here provide results on changes in healthcare seeking and health-related lifestyle during the COVID-19 lockdown in spring 2020 in Germany for the old and very old individuals from an established population-based cohort study. We also put these behavioral changes into perspective to the extent that this group had experienced infections and had been at risk for severe COVID-19 by pre-existing medical conditions irrespective of their old age. In summary, we found a majority to report no change, but almost a third with reduced healthcare seeking and a quarter with reduced physical activity. Women and the higher educated were more susceptible to change. We found no systematic changes towards increased smoking or drinking, which was supported by quantifying the change using during- and pre-lockdown reports of number of cigarettes smoked or alcoholic drinks consumed. A worsening of QOL was reported by 39%. Only 4 of the 1850 had experienced a SARS-CoV-2 infection during the first wave in spring 2020, despite a majority having sustained at least some outside contacts by continued use of public transport and doing errands themselves; 74% were at increased risk for severe COVID-19, beyond old age, by at least one medical condition listed by the CDC and WHO [5].

The coverage of old and very old aged individuals is limited in previous studies on lifestyle change during lockdown. There are notable previous efforts addressing change in smoking and drinking: an English study (in-person phone interviews, April 2020, *n* = 1674 including *n* = 389 aged 65+) [16] reported no change in smoking, but in high-risk drinking and smoking/alcohol quit attempts. It is not specified to what extent these findings also pertained to the 70+. Surveys in Italy reported decreased smoking (online, April 2020, *n* = 3522 including *n* = 120 aged 65-86 years) [17] and detailed results also on smoking quitting/relapse (online, May 2020, *n* = 6003 including *n* = 1989 aged 55-74 years) [18]. A French survey reported 27% with increased and 19% with decreased smoking, 11% with increased alcohol and 24% with decreased alcohol drinking, and increases in smoking and drinking associated with younger age (online, April 2020, *n* = 2003 including *n* = 480 aged 65+) [19]. In our AugUR survey (mailed, *n* = 1850 aged 70+, May – Aug 2020), we found no systematic change in smoking or drinking: 2% increased and 2% decreased drinking; 7% increased and 11% decreased smoking; we did not evaluate quit attempts or high-risk drinking. Compared to these published studies, ours is the largest in the 70+ aged.

Similarly, there are results on change in physical activity during lockdown: a Belgium study reported increased exercise in the young, but lower exercise among age 55-74 (online, April 2020, *n* = 13,515, including *n* = 4739 aged 55-74) [4]. In a French study, 53% reported decreased and 19% decreased physical activity, 63% increased sedentary time and 9.1% decreased (online, May 2020, *n* = 37,252) [20]; while this study included a similar sample size of the very old as ours (*n* = 670 aged 80+ versus *n* = 755 aged 80+ in AugUR), results were not specified for old or very old aged. We found 26% to report increased and 2% decreased physical activity with no difference between the 73-79 or the 80+ year-old. Older adults are prone to sedentary behavior [21] and TV watching is the most prevalent leisure-time sedentary behavior [22]. Of our participants, 15% reported increased and 2% decreased TV consumption. “Less old” age (i.e. 73-79 versus 80+), women, higher education and living alone were independently associated with increased TV consumption, which suggests a heterogeneous mix of factors influencing the changed TV consumption at lockdown – probably including a higher need for information during this early time of the pandemic as well as loneliness.

With regard to healthcare-seeking, an Australian study (online/postal invitations of volunteers, Sept 2020, *n* = 2990 in lockdown area including *n* = 414 aged 75+) [23] reported 13% with “delayed seeking of medical help”, but no specification among old aged. While timing and extent of lockdown differed between Australia and Europe, this question was comparable to our “refraining from medical appointments” answered with “yes” by 29% of our AugUR survey participants. This might reflect a different threat level between these continents. They also report on QOL aimed at physical and mental health, which cannot be compared to our assessment. Our QOL assessment at survey and prior visit was a scale from 0 (worst) to 100 (best). As Gill & Feinstein emphasized, this lacks definition of the specific QOL aspect addressed [24], but the authors also outlined the heterogeneity of > 100 QOL instruments. Given the limits on questionnaire length and our caution not to discomfort participants by un-supervised questions on anxiety and depression, we have resorted to this simple scale fully acknowledging its limitations. Nevertheless, this score reported during-lockdown (shown) and pre-lockdown (not shown, but similar) was informative, as underscored by the clear associations with covariates: decreased QOL by higher age, lower education, living alone, and pre-existing medical conditions. Interestingly, we found almost no QOL difference when comparing during- with pre-lockdown reports of the score within participants. This quantified QOL difference appears to capture a different aspect as the question “How do you consider your current (during lockdown) quality-of-life compared to before Feb 1^st^, 2020: same, better, worse”, which was answered with “worse” by 39%; more susceptible were, again, women and the higher educated, as for increased physical activity and increased TV consumption, but – interestingly – not those living alone or with pre-existing medical conditions.

Our survey is unique by the old and very old age of its participants: the 1850 AugUR COVID-19 spring 2020 survey participants were 73-98 years old at questionnaire completion. Despite that fact that this age group is most at risk for severe COVID-19 and most targeted by special shielding recommendations during lockdown, it is under-represented in other studies on lifestyle changes during lockdown. This is due to the fact that the old and very old aged are difficult to address: limited digital literacy hampers participation to online surveys; difficulty in accommodating the needs of elderly into study programs prompts most cohort studies to focus on adults aged < 70 or < 75 (e.g. NAKO [25], Gutenberg health study [26], UK Biobank [27]). For our AugUR study, we have put in extra efforts: written material in larger letters, more time per study program item, fewer items and reduced number of questions. A reason to exclude old aged is also the concern that their response is limited by comorbidities affecting physical and mental fitness. We also need to acknowledge a selection towards the more-healthy at initial baseline recruitment: baseline response was 20% [9]. This is still comparable to other cohorts’ baseline (e.g. NAKO [28]), but the usual requirements to participate in such studies, like coming to the study center, walking around within premises, and answering all questions personally, is a limiting factor at this old age and selecting towards the more-healthy. Certainly, AugUR participants are “survivors”, by having exceeded the age of 70 and – with birth years 1922 to 1947 - having grown up in between-wars and post-war Germany. To our knowledge, this is one of the largest studies on lifestyle changes during COVID-19 pandemic-related lockdown in old and very old aged individuals.

A particularly strength of our study is the very high net response of almost 90% to this COVID-19 spring 2020 survey and thus limited selection at this mailed follow-up. This can be attributed to the pronounced interest of participants to express their view in this outstanding situation, as indicated by some participants explicitly in writing. We can thus provide a relatively unselected view on the behavioral changes of AugUR cohort participants at lockdown. It is a further strength of our study that all invitees were well characterized before the pandemic from previous study center visits with regard to lifestyle factors and medical conditions. We were thus able to provide a detailed characterization of participants as well as non-responders, which is superior to convenience samples with limited control of selection. The previous visits’ assessments of lifestyle factors and QOL enabled a comparison of during-lockdown with pre-lockdown reports, rather than reporting cross-sectional prevalence in different panels of individuals, and a comparison of quantified change with perceived change. Our quantified change in physical activity, smoking and alcohol supported results from perceived change, but differed for change in QOL, as outlined above.

Our ascertainment of medical conditions via face-to-face interview, medical exams and blood draw from prior study center visits was also superior to purely relying on unsupervised questionnaires. This enabled us to characterize the proportion at increased risk for severe COVID-19 by CDC-listed medical conditions [5]. The 74% of participants at increased risk by medical conditions, beyond old age, relates well to the 73% reported for Europeans aged 70+ for a similar list of conditions using large databases from the Global Burden of Diseases, Injuries, and Risk Factors Study [29]. One U.S. survey calling random phone numbers reported 80% [30]. When extrapolating the AugUR participants’ proportion to the German population 70+ based on age- and sex-distribution, this would yield 75% in Germany. However, the generalizability is limited, due to the above noted selected towards the more healthy, and the 75% might be considered a lower bound estimate. With 13% of the German population aged 70+ and 75% at high risk within, this would indicate 10% of all Germans at high risk even when pure old age was no risk factor and no younger had any medical condition.

The fact that only four individuals of the 1850 had experienced SARS-CoV-2 infection with little to no symptoms, despite an infected as old as 95 years, is encouraging. Our observed number of infected met expectations of 4.3 infected based on health authority data in the 70+ aged inhabitants of the study region. Still, a limitation is the relying on routine targeted testing, making us – and health authorities -- miss the asymptomatic infected. Given the few infected, the 23% reporting any COVID-19 symptom since February 1st, 2020, (“cough”, “respiratory problems”, “fever”, “loss of smell”, or “loss of taste”) largely reflects the background frequency of these symptoms. Our 70+ reported symptoms less frequently than the 4684 individuals with negative SARS-CoV-2 test from NAKO [25]: 14, 8, 2, 2, 2% versus 23, 11, 8, 3, 3%, respectively. A sero-prevalence study in Tirschenreuth, Germany, found similar symptom frequencies in sero-negatives 70+ aged [31].

## Conclusion

Decreased physical activity and refraining from medical consultation of a substantial proportion of individuals are markers of collateral damage to be taken-into-account when implementing containment measures. Women and the higher educated were more susceptible and may benefit from tailored preventive measures to help maintain their usual routine. Our data suggests that the large majority of the 70+ aged are at increased risk for severe COVID-19 by pre-existing medical conditions irrespective of old age.

## Supplementary Information

**Additional file 1.**

**Additional file 2.**

## Data Availability

The datasets generated and analysed during the current study are not publicly available due to data privacy of study participants, but are available from the corresponding author on reasonable request.

## Abbreviations

AugUR study: Altersbezogene Untersuchungen zur Gesundheit der Universität Regensburg
BMI: Body mass index
CDC: Center for Disease Control and Prevention
COVID-19: Corona virus disease 2019
eGFR: Estimated glomerular filtration rate
HbA1c: Hemoglobin A1c
NAKO: NAKO Gesundheitsstudie, former: Nationale Kohorte
QOL: Quality of life
SARS-Cov-2: Severe acute respiratory syndrome coronavirus type 2
