# Supplementary material for: Changes in healthcare seeking and lifestyle in old aged individuals during COVID-19 lockdown in Germany: the population-based AugUR study
Source: BMC Geriatr. 2022 Jan 8;22:34. doi: 10.1186/s12877-021-02677-x (PMC8742665; doi:10.1186/s12877-021-02677-x)
Supplement: Supplementary file 1 — Additional file 1. [file 12877_2021_2677_MOESM1_ESM.pdf]

SUPPLEMENTARY MATERIAL

Changes in healthcare seeking and lifestyle in old aged individuals during COVID-19 lockdown in Germany: the population-based AugUR study

Brandl et al., 2021

Overview

**Supplementary Note**	Details on the AugUR COVID-19 survey questionnaire.
**Supplementary Table 1**	Proportion of participants at increased risk for severe COVID-19.
**Supplementary Table 2**	Proportions with symptoms among the 1850 participants.
**Supplementary Table 3**	Household and outside contacts among 1734 immediate responders.
**Supplementary Table 4**	Perceived changes in behavior and QOL among 1734 immediate responders.
**Supplementary Table 5A**	Influential factors for perceived changes in healthcare-seeking, lifestyle and QOL among 1850 participants.
**Supplementary Table 5B**	Influential factors for lifestyle factors and QOL at survey among the 1850 participants.
**Supplementary Table 5C**	Influential factors for quantified changes in lifestyle and QOL among the 524 survey participants with prior visit < 1 year before.

References

Supplementary Note. Details on the AugUR COVID-19 survey questionnaire.

Our questionnaire is a shortened version of the questionnaire developed by NAKO investigators, with published results by NAKO available (Peters et al., Dt. Ärzteblatt 2021). We shortened the questionnaire to meet the needs of the elderly by larger letters, less items, excluding any questions on occupation and unemployment, and by avoiding unsupervised questions on anxiety or depression. The questionnaire targeted the following: (i) testing and infection: SARS-CoV-2 testing (status, time/location, reason); COVID-19 related symptoms (cough, shortness of breath, respiratory problems, fever, chills, loss of smell/taste, bronchitis/pneumonia); other symptoms related to general infections (pain in extremities, diarrhea, nausea, red eye/eye infection, headache, fatigue, rhinitis); hospital stay potentially linked to COVID-19 (bronchitis/pneumonia); (ii) living situation: household (living alone, ≥ 1 other person, nursing home; age and relationship to household members); potential exposure from outside contacts (contact to infected individual < 1.5m and > 15 min, use of public transport, conduct of errands, help come to household) or indirect outside contacts via a younger generation in the household (i.e. person <50 years of age[1]); (iii) refraining from medical consultation (i.e. cancelling routine appointments or postponing appointments despite acute need); (iv) lifestyle factors and QOL during the lockdown: smoking (status as current, former, never; number of cigarettes smoked per day); alcohol consumption (frequency of drinking, number of drinks typically consumed; one drink defined as a small bottle of beer, 0,33l, a small glass of wine, 0,125l, or liquor, 4cl.); TV consumption (days per week with TV consumption for >2 hours); physical activity as categories of weekly hours of light activity (0, 0-2 hours, >2 hours; including bicycling, gardening, walking); QOL on a scale from 0 (very poor) to 100 (excellent); (iv) perceived changes in lifestyle factors and QOL when compared to before the corona pandemic (as per Feb 1st, 2020). The full English version of the questionnaire can be found as an **Appendix.**

Supplementary Table 1. Proportion of participants at increased risk for severe COVID-19. Medical conditions of AugUR study platform participants eligible for this AugUR COVID-19 survey were derived at the prior study center visit 0-48 months before lockdown from medical exams, serum measurements, and face-to-face interview. For the 1850 survey participants, we show the proportion at increased risk or possibly increased risk for severe COVID-19 (i.e. requiring hospitalization, intensive care, ventilator, or death) as defined by the Center of Disease Control.[2]

**Participants**	**All n=1850**	**Women n=972**	**Men n=878**	**Age at prior visit 70-79 n=1095**	**Age at prior visit 80+ n=755**
**In the study**					
**Any increased risk**[a,], % (n)	**74.3 (1368)**	**72.4 (699)**	**76.5 (669)**	**70.8 (773)**	**79.5 (595)**
Cancer[b], % (n)	26.3 (485)	23.3 (226)	29.6 (259)	25.2 (275)	27.9 (210)
Chronic kidney disease[c], % (n)	27.9 (512)	28.1 (269)	27.7 (243)	21.4 (233)	37.6 (279)
Chronic bronchitis, % (n)	6.4 (118)	6.3 (61)	6.5 (57)	5.2 (57)	8.1 (61)
Obesity[d], % (n)	30.1 (556)	31.1 (302)	29.0 (254)	30.3 (332)	29.7 (224)
Serious heart conditions[e], % (n)	27.1 (496)	22.1 (212)	32.5 (284)	22.7 (246)	33.4 (250)
Type 2 diabetes[f], % (n)	20.7 (382)	18.5 (179)	23.2 (203)	19.5 (213)	22.4 (169)
**Any possibly increased risk**[a] , % (n)	**84.8 (1561)**	**82.9 (799)**	**87.0 (762)**	**85.1 (928)**	**84.4 (633)**
Asthma (moderate to severe), % (n)	6.9 (128)	7.5 (73)	6.3 (55)	7.9 (86)	5.6 (42)
Hypertension[f], % (n)	70.3 (1297)	70.6 (683)	70.0 (614)	68.1 (744)	73.4 (553)
Cerebrovascular disease[g], % (n)	8.7 (159)	6.2 (60)	11.3 (99)	7.7 (84)	10.0 (75)
Current/former smoker[h], % (n)	42.9 (791)	29.0 (280)	58.3 (511)	47.6 (520)	36.1 (271)
**Any of the above**, % (n)	**93.6 (1728)**	**92.1 (863)**	**94.9 (819)**	**93.1 (1018)**	**94.4 (710)**

Abbreviations: T2DM = type 2 diabetes; [a]) As defined by CDC[2]. [b]) Excluding white skin cancer. [c]) Chronic kidney disease defined as estimated glomerular filtration rate based on serum creatinine < 60 mg/dl/1.73m². [d]) Defined as BMI ≥ 30. [e]) Self-reported history of myocardial infarction OR percutaneous coronary intervention OR coronary bypass surgery. [f]) Self-reported diagnosis. [g]) Self-reported diagnosis of stroke. [h]) Currently smoking ≥1 cigarette per day or having stopped smoking for ≥1 month.

Supplementary Table 2. Proportions with symptoms among the 1850 participants. Shown is the proportion of 1850 AugUR COVID-19 survey participants who reported symptoms related to COVID-19 or more generally to infections (via self-completion questionnaire).

Symptom	Overall n=1850	Women n=972	Men n=878	Age at survey 73-79 n=829	Age at survey 80+ n=1021
**Related to COVID-19**					
Cough, % (n)	14.4 (266)	13.7 (133)	15.2 (133)	14.9 (123)	14.0 (143)
Shortness of breath, % (n)	5.3 (97)	4.8 (47)	5.7 (50)	4.7 (39)	5.7 (58)
Respiratory problems, % (n)	7.6 (141)	7.7 (75)	7.6 (66)	7.6 (63)	7.7 (78)
Fever, % (n)	1.8 (34)	2.0 (19)	1.7 (15)	1.6 (13)	2.1 (21)
Chills, % (n)	1.8 (34)	1.9 (18)	1.8 (16)	1.3 (11)	2.3 (23)
Loss of smell, % (n)	1.7 (32)	1.5 (15)	1.9 (17)	1.6 (13)	1.9 (19)
Loss of taste, % (n)	1.7 (32)	2.0 (19)	1.5 (13)	1.6 (13)	1.9 (19)
**At least one of the above, % (n)**	**22.8 (421)**	**22.3 (217)**	**23.3 (204)**	**22.8 (188)**	**22.9 (233)**
**Related to infections**					
Red eye/eye infection, % (n)	7.0 (129)	8.0 (78)	5.8 (51)	6.2 (51)	7.7 (78)
Limb pain, % (n)	17.1 (315)	18.5 (180)	15.4 (135)	14.6 (121)	19.0 (194)
Diarrhea, % (n)	6.5 (120)	7.5 (73)	5.4 (47)	6.1 (50)	6.9 (70)
Nausea, % (n)	3.0 (55)	3.8 (37)	2.1 (18)	2.4 (20)	3.4 (35)
Head ache, % (n)	8.3 (153)	11.0 (107)	5.3 (46)	9.8 (81)	7.1 (72)
Fatigue, % (n)	19.6 (362)	20.8 (202)	18.3 (160)	17.1 (141)	21.7 (221)
Rhinitis, % (n)	13.3 (246)	11.9 (116)	14.9 (130)	13.1 (108)	13.5 (138)
**At least one of the above, % (n)**	**41.2 (761)**	**44.6 (433)**	**37.5 (328)**	**38.4 (317)**	**43.6 (444)**
**Bronchitis/pneumonia, any, % (n)**	**6.6 (121)**	**6.7 (64)**	**6.6 (57)**	**5.7 (47)**	**7.4 (74)**
Mild symptoms, % (n)	4.2 (77)	4.5 (43)	3.9 (34)	3.7 (30)	4.7 (47)
Bed ridden, % (n)	0.2 (4)	0.4 (4)	0.0 (0)	0.2 (2)	0.2 (2)
Requiring physician, % (n)	1.4 (26)	1.1 (11)	1.7 (15)	1.3 (11)	1.5 (15)
Hospitalized, % (n)	0.8 (14)	0.6 (6)	0.9 (8)	0.5 (4)	1.0 (10)
**Any of the above, % (n)**	**48.0 (881)**	**50.9 (493)**	**44.8 (388)**	**45.1 (372)**	**50.3 (509)**

Multiple answers possible, except for severity of bronchitis/pneumonia.

Supplementary Table 3. Household and outside contacts among 1734 immediate responders. Shown is the household situation and some aspects of behavior involving outside contact for the 1734 immediate responders as per questionnaire completion (questionnaire return May 13th to June 12th, 2020).

**Living situation, outside contacts**	**Overall n=1734**	**Women n=901**	**Men n=878**
**Household**			
Living alone, % (n)	35.2 (602)	49.4 (440)	19.8 (162)
Living with ≥ 1 person, % (n)	63.6 (1087)	49.3 (439)	79.2 (648)
Living in a nursing home, % (n)	1.1 (29)	1.2 (11)	1.0 (8)
**Outside contacts**			
Contact with infected person[b], % (n)	1.0 (17)	0.7 (6)	1.4 (11)
Living with younger generation person[a], % (n)	2.6 (45)	2.0 (18)	3.3 (27)
Using public transport[c], % (n)	24.7 (420)	29.3 (260)	19.6 (160)
Doing errands[c], % (n)	81.3 (1395)	80.7 (722)	82.1 (673)
Having a help come to the household[c], % (n)	17.4 (294)	18.9 (165)	15.8 (129)

[a]) Defined as additional person in household with < 50 years of age. [b]) Contact for more than 15 minutes at a distance less than 1.5 meter or person in the same household. [c]) Defined as ever using public transport / ever doing errands / ever having a help come to the household during February 1st until July 12th, 2020.
Available n (overall, women, men): Contact with infected person 1669 / 857 / 812; Living with younger generation person 1708 / 890 / 818; Using public transport 1702 / 886 / 816; Doing errands 1715 / 895 / 820; Having a help come to the household 1694 / 875 / 819;

Supplementary Table 4. Perceived changes in behavior and QOL among 1734 immediate responders. Shown are the perceived changes as per questionnaire completion since Feb 1st, 2020 by the 1734 immediate responders (questionnaire return May 13th to June 12th, 2020).

Perceived changes (now vs. before corona crisis)	Overall[a] n=1734	Women n=901	Men n=833	Age at survey 73-79 n=789	Age at survey 80+ n=945
**Refraining from medical consultation**					
No, % (n)	71.0 (1190)	67.7 (584)	74.6 (606)	71.3 (547)	70.8 (643)
Yes, % (n)	29.0 (485)	32.3 (279)	25.4 (206)	28.7 (220)	29.2 (265)
Rescheduling, % (n)	21.7 (363)	23.4 (202)	19.8 (161)	22.8 (175)	20.7 (188)
Despite acute need, % (n)	0.9 (15)	1.2 (10)	0.6 (5)	0.9 (7)	0.9 (8)
Despite regular, % (n)	6.4 (107)	7.8 (67)	4.9 (40)	5.0 (38)	7.6 (69)
**Using public transport**					
Less, % (n)	33.4 (524)	41.0 (331)	25.4 (193)	32.5 (238)	34.2 (286)
Same, % (n)	63.2 (992)	54.8 (443)	72.1 (549)	64.5 (473)	62.1 (519)
More, % (n)	3.4 (53)	4.2 (34)	2.5 (19)	3.0 (22)	3.7 (31)
**Doing errands**					
Less, % (n)	42.5 (720)	51.1 (450)	47.5 (270)	43.1 (332)	42.1 (388)
Same, % (n)	56.1 (949)	47.9 (422)	92.6 (527)	55.3 (426)	56.7 (523)
More, % (n)	1.4 (24)	1.0 (9)	2.6 (15)	1.7 (13)	1.2 (11)
**Getting food delivered**					
Less, % (n)	0.8 (9)	1.1 (6)	0.5 (3)	0.6 (3)	1.0 (6)
Same, % (n)	95.9 (1096)	95.7 (513)	96.0 (583)	98.0 (528)	94.0 (568)
More, % (n)	3.3 (38)	3.2 (17)	3.5 (21)	1.5 (8)	5.0 (30)
**Physical activity[b]**					
Less, % (n)	26.5 (439)	31.7 (271)	20.9 (168)	25.3 (193)	27.5 (246)
Same, % (n)	71.3 (1180)	66.0 (564)	76.8 (616)	71.8 (548)	70.8 (632)
More, % (n)	2.2 (37)	2.2 (19)	2.2 (18)	2.9 (22)	1.7 (15)
**TV consumption**					
More, % (n)	14.8 (248)	18.5 (161)	10.8 (87)	16.1 (124)	13.7 (124)
Same, % (n)	83.4 (1399)	80.0 (697)	87.0 (702)	82.3 (634)	84.3 (765)
Less, % (n)	1.8 (31)	1.5 (13)	2.2 (18)	1.6 (12)	2.1 (19)
**Smoking[c]**					
More, % (n)	8.0 (4)	11.5 (3)	4.2 (1)	12.5 (4)	0.0 (0)
Same, % (n)	80.0 (40)	76.9 (20)	85.2 (20)	71.9 (23)	94.4 (17)
Less, % (n)	12.0 (6)	11.5 (3)	12.5 (3)	15.6 (5)	5.6 (1)
**Alcohol consumption[d]**					
More, % (n)	2.8 (37)	2.4 (15)	3.2 (22)	2.9 (18)	2.7 (19)
Same, % (n)	95.0 (1268)	96.2 (614)	93.8 (654)	94.2 (590)	95.6 (678)
Less, % (n)	1.8 (30)	1.4 (9)	3.0 (21)	2.9 (18)	1.7 (12)
**Perceived QOL**					
Worse, % (n)	38.5 (631)	41.1 (347)	35.7 (284)	39.9 (302)	37.3 (329)
Same, % (n)	61.3 (1004)	58.6 (495)	64.0 (509)	59.9 (453)	62.4 (551)
Better, % (n)	0.2 (4)	0.2 (2)	0.3 (2)	0.1 (1)	0.3 (3)

[a]) n is different for each variable (n=sum of the respective rows). [b]) Including bicycling, gardening, walking. [c]) Among current smoker as per survey (n=5054), defined as currently smoking ≥1 cigarette per day. [d]) For individuals with any alcohol consumption during the last 12 months (as per survey, n=13351424).

Supplementary Table 5A. Influential factors for perceived changes in healthcare-seeking, lifestyle and QOL among 1850 participants. Shown are Odd Ratio (OR) estimates and P-values from logistic regression for perceived changes in healthcare-seeking, lifestyle factors and QOL as outcome as per questionnaire completion (May 13th to Aug 26th, 2020) since Feb 1st, 2020. Also shown are Prob(Y=1|X=0) [exp(intercept) and P-value]. We did not evaluate perceived change of smoking or drinking, due to a lack of trend towards decrease or increase and only few individuals reporting any change. Three nested models were computed (Model I, II, III; n given for analyzed sample in model III). P-values < 0.05 are bold. For Model I, we evaluated age-by-sex interaction, for Model II, sex-by-education interaction: no interaction showed P-value < 0.05.

Perceived change	Model I	Model II	Model III
**Refrained from medical consultation [0=no, 1=yes] (n=1668)**	**OR (P)**	**OR (P)**	**OR (P)**
Intercept	[0.471 (<0.001)]	[0.424 (<0.001)]	[0.383 (<0.001)]
Age[a] [years]	1.002 (0.864)	1.001 (0.906)	1.000 (0.983)
Sex [0=women, 1=men]	**0.724 (0.003)**	**0.725 (0.007)**	**0.712 (0.005)**
Education[b] [years]	NA	1.023 (0.155)	1.025 (0.122)
Living alone[c] [0=no, 1=yes]	NA	1.129 (0.307)	1.126 (0.317)
At increased risk[d] [0=no, 1=yes]	NA	NA	1.156 (0.252)
**More TV consumption [0=no, 1=yes] (n=1754)**	**OR (P)**	**OR (P)**	**OR (P)**
Intercept	[0.227 (<0.001)]	[0.140 (<0.001)]	[0.160 (<0.001)]
Age[a] [years]	**0.955 (0.003)**	**0.948 (0.001)**	**0.950 (0.001)**
Sex [0=women, 1=men]	**0.540 (<0.001)**	**0.585 (0.001)**	**0.592 (0.001)**
Education[b] [years]	NA	**1.057 (0.006)**	**1.054 (0.010)**
Living alone[c] [0=no, 1=yes]	NA	**2.016 (<0.001)**	**2.029 (<0.001)**
At increased risk[d] [0=no, 1=yes]	NA	NA	0.837 (0.249)
**Perceived decrease in physical activity [0=no, 1=yes] (n=1732)**	**OR (P)**	**OR (P)**	**OR (P)**
Intercept	[0.438 (<0.001)]	[0.374 (<0.001)]	[0.335 (<0.001)]
Age[a] [years]	1.003 (0.779)	1.001 (0.917)	1.001 (0.964)
Sex [0=women, 1=men]	**0.593 (<0.001)**	**0.598 (<0.001)**	**0.586 (<0.001)**
Education[b] [years]	NA	1.032 (0.058)	**1.034 (0.043)**
Living alone[c] [0=no, 1=yes]	NA	1.230 (0.084)	1.225 (0.090)
At increased risk[d] [0=no, 1=yes]	NA	NA	1.175 (0.215)
**Perceived worsening in QOL [0=no, 1=worse] (n=1707)**	**OR (P)**	**OR (P)**	**OR (P)**
Intercept	[0.699 (<0.001)]	[0.583 (<0.001)]	[0.563 (<0.001)]
Age[a] [years]	0.982 (0.084)	0.980 (0.064)	**0.978 (0.046)**
Sex [0=women, 1=men]	**0.810 (0.033)**	**0.746 (0.008)**	**0.736 (0.006)**
Education[b] [years]	NA	**1.063 (<0.001)**	**1.063 (<0.001)**
Living alone[c] [0=no, 1=yes]	NA	1.151 (0.201)	1.154 (0.197)
At increased risk[d] [0=no, 1=yes]	NA	NA	1.055 (0.643)

[a]) For these analyses age - 80 was used. [b]) Counting years of school, vocational school, and/or university including PhD time, if applicable; for these analyses "education -10 years" was used. [c]) No other persons in the household. [d]) Any medical condition listed by CDC[2] for increased risk for severe COVID-19.

Supplementary Table 5B. Influential factors for lifestyle factors and QOL reported at survey among the 1850 participants. Shown are beta / Odd Ratio estimates and P-values from linear/logistic regression with lifestyle factor or QOL as outcome variable. Lifestyle factors and QOL here were reported at survey (questionnaire completion May 13th to Aug 26th). Also shown are Prob(Y=1|X=0) [exp(intercept) and P-value]. Three different nested models were computed (Model I, II, III; n given for analyzed sample in model III). P-values < 0.05 in bold. For Model I, we evaluated age-by-sex interaction, for Model II, sex-by-education interaction: some interactions were found with P-value < 0.05 and were indicated in bold.

Factor reported at survey	Model I	Model II	Model III
**High physical activity at survey [0=no, 1=yes] (n=1783)**	**OR (P)**	**OR (P)**	**OR (P)**
Intercept	[1.436 (<0.001)]	[1.377 (<0.001)]	[2.223 (<0.001)]
Age[a] [years]	**0.925 (<0.001)**	**0.928 (<0.001)**	**0.932 (<0.001)**
Sex [0=women, 1=men]	**1.544 (<0.001)**	**1.302 (0.017)**	**1.364 (0.006)**
Education[b] [years]	NA	**1.066 (<0.001)**	**1.059 (<0.001)**
Living alone[c] [0=no, 1=yes]	NA	0.941 (0.577)	0.967 (0.761)
At increased risk[d] [0=no, 1=yes]	NA	NA	**0.518 (<0.001)**
**Smoking at survey (among smokers at survey, n=48) [# cigs. per day]**	**beta (P)**	**beta (P)**	**beta (P)**
Intercept	[7.647 (<0.001)]	[6.540 (<0.001)	[4.800 (0.014)]
Age[a] [years]	**-0.491 (0.026)**	**-0.650 (0.003)**	**-0.659 (0.003)**
Sex [0=women, 1=men]	3.486 (0.074)	**4.520 (0.018)**	**4.417 (0.020)**
Education[b] [years]	NA	-0.547 (0.073)	-0.565 (0.064)
Living alone[c] [0=no, 1=yes]	NA	3.256 (0.074)	2.998 (0.099)
At increased risk[d] [0=no, 1=yes]	NA	NA	3.040 (0.099)
**Alcohol at survey (among consumers at survey, n=1276) [#drinks per day]**	**beta (P)**	**beta (P)**	**beta (P)**
Intercept	[0.680 (<0.001)]	[0.639 (<0.001)	[0.725 (<0.001)]
Age[a] [years]	**-0.016 (0.007)**	**-0.014 (0.024)**	**-0.013 (0.044)**
Sex [0=women, 1=men]	**0.554 (<0.001)**	**0.500 (<0.001)**	**0.511 (<0.001)**
Education[b] [years]	NA	**0.025 (0.002)**	**0.024 (0.004)**
Living alone[c] [0=no, 1=yes]	NA	-0.007 (0.905)	-0.004 (0.951)
At increased risk[d] [0=no, 1=yes]	NA	NA	**-0.123 (0.050)**
**QOL at survey [# score points] (n=1726)**	**beta (P)**	**beta (P)**	**beta (P)**
Intercept	[68.773 (<0.001)]	[69.445 (<0.001)]	[72.955 (<0.001)]
Age[a] [years]	**-0.596 (<0.001)**	**-0.536 (<0.001)**	**-0.497 (<0.001)**
Sex [0=women, 1=men]	**2.657 (0.004)**	0.947 (0.356)	1.152 (0.261)
Education[b] [years]	NA	**0.399 (0.004)**	**0.363 (0.009)**
Living alone[c] [0=no, 1=yes]	NA	**-2.522 (0.015)**	**-2.480 (0.016)**
At increased risk[d] [0=no, 1=yes]	NA	NA	**-4.793 (<0.001)**

[a]) For these analyses age -80 was used. [b]) Counting years of school, vocational school, and/or university including PhD time, if applicable; for these analyses "education -10 years" was used. [c]) No other persons in the household. [d]) Any medical condition in the list of the CDC[2] for increased risk for severe COVID-19.

Supplementary Table 5C. Influential factors for quantified changes in lifestyle and QOL among the 524 survey participants with prior visit < 1 year before. Shown are beta- / Odd Ratio estimates and P-values from linear/logistic regression for quantified changes in lifestyle factors and QOL as outcome variables. Also shown are Prob(Y=1|X=0) [exp(intercept) and P-value]. Quantified change in smoking was not analyzed due to scarce numbers of smokers among the 524 (n=14). Quantified changes are the difference of reported values at survey compared to reported values at prior visit. This analysis is restricted to the 524 individuals with prior visit < 1 year before lockdown (between March 2019 and March 2020). Three different nested models were computed (Model I, II, III; n given for analyzed sample in model III). P-values < 0.05 in bold. For Model I, we evaluated age-by-sex interaction, for Model II, sex-by-education interaction: no interaction showed P-value < 0.05.

Quantified change	Model I	Model II	Model III
**Quantified lower category in physical activity [0=not a lower category, 1=a lower category] (n=502)**	**OR (P)**	**OR (P)**	**OR (P)**
Intercept	[0.266 (<0.001)]	[0.266 (<0.001)]	[0.249 (<0.001)]
Age[a] [years]	1.021 (0.397)	1.031 (0.230)	1.032 (0.220)
Sex [0=women, 1=men]	0.682 (0.100)	0.631 (0.066)	0.629 (0.062)
Education[b] [years]	NA	1.033 (0.329)	1.034 (0.309)
Living alone[c] [0=no, 1=yes]	NA	0.838 (0.482)	0.840 (0.488)
At increased risk[d] [0=no, 1=yes]	NA	NA	1.112 (0.686)
**Quantified change in alcohol, i.e. difference in # drinks after vs. before [#drinks] (n=437)**	**beta (P)**	**beta (P)**	**beta (P)**
Intercept	[0.039 (0.426)]	[0.024 (0.719)]	[0.020 (0.810)]
Age[a] [years]	**-0.027 (0.001)**	**-0.028 (<0.001)**	**-0.028 (<0.001)**
Sex [0=women, 1=men]	0.054 (0.441)	0.094 (0.221)	0.098 (0.209)
Education[b] [years]	NA	-0.010 (0.320)	-0.010 (0.320)
Living alone[c] [0=no, 1=yes]	NA	0.074 (0.348)	0.076 (0.341)
At increased risk[d] [0=no, 1=yes]	NA	NA	0.002 (0.982)
**Quantified change in QOL, i.e. difference in QOL scores after vs. before [#score points] (n=471)**	**beta (P)**	**beta (P)**	**beta (P)**
Intercept	[-2.149 (0.103)]	[-0.127 (0.942)]	[0.408 (0.860)]
Age[a] [years]	0.048 (0.818)	0.114 (0.590)	0.126 (0.556)
Sex [0=women, 1=men]	1.083 (0.569)	0.525 (0.798)	0.365 (0.859)
Education[b] [years]	NA	-0.210 (0.446)	-0.213 (0.440)
Living alone[c] [0=no, 1=yes]	NA	-3.153 (0.139)	-3.344 (0.118)
At increased risk[d] [0=no, 1=yes]	NA	NA	-0.447 (0.835)

[a]) For these analyses age -80 was used. [b]) Counting years of school, vocational school, and/or university including PhD time, if applicable; for these analyses "education -10 years" was used. [c]) No other persons in the household. [d]) Any medical condition in the list of the CDC[2] for increased risk for severe COVID-19.

REFERENCES

1. Brandén M, Aradhya S, Kolk M, Härkönen J, Drefahl S, Malmberg B, et al. Residential Context and COVID-19 Mortality among the Elderly in Stockholm : A population-based , observational study Residential Context and COVID-19 Mortality among the Elderly in Stockholm : 2020.

2. CDC. Available from: https://www.cdc.gov/coronavirus/2019-ncov/need-extra-precautions/evidence-table.html
