# Supplementary material for: Changes in healthcare seeking and lifestyle in old aged individuals during COVID-19 lockdown in Germany: the population-based AugUR study
Source: BMC Geriatr. 2022 Jan 8;22:34. doi: 10.1186/s12877-021-02677-x (PMC8742665; doi:10.1186/s12877-021-02677-x)
Supplement: Supplementary file 2 — Additional file 2. [file 12877_2021_2677_MOESM2_ESM.pdf]

AugUR Questionnaire about the coronavirus pandemic

Dear participant,

We very much appreciate your support by filling out this questionnaire. The term „coronavirus pandemic“ used in the following describes the current outbreak of infections with the novel coronavirus (SARS-CoV-2).

All of your answers are treated with absolute confidentiality and are exclusively evaluated in the course of this study, without reference to your name. Results are exclusively evaluated by taking together the answers of all participants as average values or percentages.

Please send the completed questionnaire back postage paid to the AugUR study centre within the attached envelope. We are taking care of the postage for you.

Please bear in mind the following instructions about how to fill out the questionnaire correctly:

• Please use a **black or blue pen.**	
• For **checkboxes** without further indications, please tick **exactly one** answer. Questions that allow more than one answer are indicated as such at the end of the question.<br>In case you want to change an already ticked answer, please cross out the old answer in a clearly visible manner and tick the more accurate answer.	⊗ Yes ○ No<br><br>⊗ Yes ~~⊗ No~~
• Please write **numbers** into the provided boxes.<br>In case you want to change a number, please cross out the old number and write down the correct number above the one you crossed out.	Year of diagnosis 2 0 1 5<br><br>2 0 1 7<br>Year of diagnosis ~~2 0 1 5~~
• Please skip questions only if indicated next to the question.	→ Please continue with **question 4**

AugUR Questionnaire about the coronavirus pandemic

The current date

☐☐/☐☐/2020
day month

1. How would you rate your state of health?

○ 1 Very good

○ 2 Good

○ 3 Satisfactory

○ 4 Sufficient

○ 5 Poor

○ 6 Unsatisfactory

2. How many persons are currently living in your household?

○ more than 1 person (spouse/ life companion, family, friends, shared accomodation)

○ I live alone. **->** *Please continue with **question 4***

○ I live alone in a residential community (assisted living, retirement home, nursing home etc.). **->** *Please continue with **question 4***

3. In case there are currently more than one person living in your household (meaning: yourself and at least one other person), please describe the current members of your household in the following table.

A household is defined as a group of people, with whom you are living together or mostly living together (spouse/ life companion, family, shared accommodation).

Please indicate the age „0“ in the column for “age” for infants younger than one year.

	**Age**	**Gender**		**Relationship to yourself**			
	(in years)	Female	Male	Spouse/ life companion	Daughter/ son	Grandchild	Other
1. Person (yourself)		☐	☐				
2. Person		☐	☐	☐	☐	☐	☐
3. Person		☐	☐	☐	☐	☐	☐
4. Person		☐	☐	☐	☐	☐	☐
5. Person		☐	☐	☐	☐	☐	☐
6. Person		☐	☐	☐	☐	☐	☐
7. Person		☐	☐	☐	☐	☐	☐
8. Person		☐	☐	☐	☐	☐	☐
9. Person		☐	☐	☐	☐	☐	☐
10. Person		☐	☐	☐	☐	☐	☐

4. Since February the 1st 2020, have you ever suffered from one of the following symptoms?
Please tick all of the symptoms you have experienced.

- ☐ cough
- ☐ shortness of breath
- ☐ difficulty breathing
- ☐ fever
- ☐ chills
- ☐ limb pain
- ☐ diarrhea
- ☐ smell disorders
- ☐ taste disorders
- ☐ nausea
- ☐ eye inflammation
- ☐ headache
- ☐ fatigue
- ☐ cold
- ☐ other

5. Since February the 1st 2020, has anybody else within your household suffered from one of the following symptoms? Please tick all of the symptoms that at least one of the other persons within your household has experienced.

- ☐ cough
- ☐ shortness of breath
- ☐ difficulty breathing
- ☐ fever
- ☐ chills
- ☐ limb pain
- ☐ diarrhea
- ☐ smell disorders
- ☐ taste disorders
- ☐ nausea
- ☐ eye inflammation
- ☐ headache
- ☐ fatigue
- ☐ cold
- ☐ other

6. Since the February the 1st 2020, have you ever suffered from bronchitis or pneumonia?

- ○ No.
- ○ Yes, I was slightly suffering from these conditions.
- ○ Yes, due to these conditions, I had to stay in bed and was incapable of going on with my normal daily routine (house work, hobbies). However, I did not seek medical support.
- ○ Yes, due to these conditions, I visited a doctor/ sent for a doctor/ received ambulatory treatment in the hospital (without overnight stay).
- ○ Yes, due to these conditions, I received stationary treatment in the hospital (at least one overnight stay).

7. Since February the 1st 2020, has anybody else within your household suffered from bronchitis or pneumonia?

○ No.

○ Yes, the other person(s) were slightly suffering from these conditions.

○ Yes, due to these conditions, they had to stay in bed, and were incapable of going on with their normal daily routine (house work, hobbies). However, they did not seek medical support.

○ Yes, due to these conditions, the other person(s) visited a doctor/ sen for a doctor/ received ambulatory treatment in the hospital (without an overnight stay).

○ Yes, due to these conditions, the other person(s) received stationary treatment in the hospital (at least one overnight stay).

8. Have you ever been tested for the coronavirus (once or several times) in a doctor´s practice/ test centre/ hospital?

○ Yes. ○ No. **->** *Please continue with* ***question 11***

↓

Has there ever been detected a coronavirus infection (test result positive)?

○ Yes. ○ No.

9. When have you been tested for the coronavirus?
In case you were tested positive, please indicate the date of your first positive coronavirus test. In case you were always tested negative, please indicate the date of your first coronavirus test.

Please indicate the date here: ☐☐/☐☐/2020

10. Why were you tested for the coronavirus?
It is possible to name more than one reason.

○ I had contact to a person, who was infected with the coronavirus or who was suspected to be infected.

○ I returned from a risk area 14 days prior to the coronavirus test.

○ I had symptoms, which are associated with a coronavirus infection, at the time of the test. -> Please check all of the symptoms you experienced at the time of your test.

☐ cough	☐ shortness of breath	☐ difficulty breathing
☐ fever	☐ chills	☐ limb pain
☐ diarrhea	☐ smell disorders	☐ taste disorders
☐ nausea	☐ eye inflammation	☐ headache
☐ fatigue	☐ cold	☐ others

○ None of the named reasons apply.

11. Has another person within your household been tested for the coronavirus (once or several times)?

○ Yes. ○ No. **->** *Please continue with* ***question 12***

↓

If so, how many persons have been tested?

number of adults: _____ number of children (< 18 years): _____

Has there been detected a coronavirus infection (test result positive)?

○ Yes. ○ No.

↓

If so, for how many persons has an infection been detected?

number of adults: _____ number of children (< 18 years): _____

12. Since February the 1st 2020, have you been in contact with a person, who was infected at this time or who was diagnosed with a coronavirus infection within 14 days after the contact, for more than 15 minutes (e.g. a longer conversation, a shared meal, various short conversations)?

○ Yes, one person within my household.

○ Yes, one person outside my household with a distance less than 1,5 meter.

○ Yes, one person outside my household with a distance more than 1,5 meter.

○ No.

13. Have you been more reluctant than usual to visit a doctor at any time during the coronavirus pandemic?

○ No, my behavior regarding this matter did not change.

○ Yes, I avoided doctor´s appointment at least once, which I would have sought otherwise. I postponed the doctor´s appointment.

○ Yes, I avoided a doctor´s appointment at least once, although I was feeling severely ill.

○ I avoided regular doctor's appointments, which I would have sought otherwise.

14. How would you rate your current quality of life?

Please state a number between 0 (very bad) and 100 (very good):

☐☐☐

Did your quality of life change during the coronavirus pandemic?

○ It remained the same.

○ It used to be better.

○ It used to be worse.

15. Are you currently using public transportation (e.g. bus, train)?

○ Yes, daily or more than three times a week.
○ Yes, between one and three times a week.
○ Yes, less than once a week.
○ No, never or hardly ever.

Has your behavior (regarding this topic) changed during the coronavirus pandemic?

○ It used to be more frequent.
○ It remained the same.
○ It used to be less frequent.

16. How often are you watching television for two or more hours, without a break?

○ Daily or more than three times a week.
○ Between one and three times a week.
○ Less than one time a week.
○ Never or hardly ever.

Has your behavior (regarding this topic) changed during the coronavirus pandemic?

○ It used to be more frequent.
○ It remained the same.
○ It used to be less frequent.

17. How often are you exercising? Bicycling, gardening and going for a walk also count as exercise.

○ Frequently for more than four hours a week.

○ Frequently for between two and four hours a week.

○ Frequently for between one and two hours a week.

○ Less than one hour a week.

○ I do not exercise.

Has your behavior (regarding this topic) changed during the coronavirus pandemic?

○ It used to be more frequent.

○ It remained the same.

○ It used to be less frequent.

18. Are you currently shopping for groceries yourself?

○ Daily or more than three times a week.

○ Between one and three times a week.

○ Less than one time a week.

○ Never or hardly ever.

Has your behavior (regarding this topic) changed during the coronavirus pandemic?

○ It used to be more frequent.

○ It remained the same.

○ It used to be less frequent.

19. Are you currently having food delivered to you?

○ Daily or more than three times a week.

○ Between one and three times a week.

○ Less than one time a week.

○ Never or hardly ever.

Has your behavior (regarding this topic) changed during the coronavirus pandemic?

○ It used to be more frequent.

○ It remained the same.

○ It used to be less frequent.

○ It used to be never.

20. Is a housekeeper currently helping you?

○ Daily or more than three times a week.

○ Between one and three times a week.

○ Less than one time a week.

○ Never or hardly ever.

Has your behavior (concerning this topic) changed during the corona-crisis?

○ It used to be more frequent.

○ It remained the same.

○ It used to be less frequent.

○ It never occurred.

21. How often are you currently drinking an alcoholic beverage (e.g. a glass of wine, beer, liqueur, schnapps)?

○ Never. -> *Please continue with* ***question 23***

○ Once a month or less frequent.

○ Between two and four times a month.

○ Between two and four times a week.

○ Between five to six times per week.

○ Once a day or more frequent.

22. If you are having a drink, how many alcoholic beverages do you usually consume on average <u>at this particular day</u>? One alcoholic beverage corresponds to one small bottle of beer (0,33l), one small glass of wine (0,125l) or two shots of schnapps (4cl).

○ Between one and two alcoholic beverages.

○ Between three and four alcoholic beverages.

○ Between five and six alcoholic beverages.

○ Between seven and nine alcoholic beverages.

○ Ten or more alcoholic beverages.

Has your behavior (regarding this topic) changed during the coronavirus pandemic?

○ I used to drink more alcohol.

○ I used to drink the same amount of alcohol.

○ I used to drink less alcohol.

○ I used to never drink alcohol.

23. Are you currently smoking cigarettes, cigars, small cigars, pipe, or any other products containing tobacco?

○ No, I am currently not smoking and I have never smoked. -> *you reached* **the End**

○ No, I am currently not smoking, but I used to smoke in the past.
Please state when you stopped smoking (age!): ☐☐ years old

○ Yes.

↓

What are you smoking and how much are you currently smoking on average <u>per day</u>?

cigarettes ☐☐ pieces a day

pipe ☐☐ times a day

cigars/small cigars ☐☐ pieces a day

Has your behavior (regarding this topic) changed during the coronavirus pandemic?

○ I used to smoke more.

○ I used to smoke the same amount.

○ I used to smoke less.

○ I used to never smoke.

THE END*

You made it! Thank you very much for your support.

Please put the questionnaire in the attached, postage free return envelope and throw it into the letter box.

Much obliged!

****
